# A vicious cycle of microglial dysfunction: bridging synaptic pruning and neuroinflammation across the neurodevelopmental continuum

**DOI:** 10.3389/fimmu.2026.1883650

**Published:** 2026-08-06

**Authors:** Fanqian Yang, Qing Tang, Xiaofang Wang, Jinyu Chen, Yuxin Li, Wenbo Liu, Bingxiang Ma, Wenli Shi

**Affiliations:** 1 Pediatrics Hospital, The First Affiliated Hospital of Henan University of Chinese Medicine, Zhengzhou, Henan, China; 2 School of Pediatrics, Henan University of Chinese Medicine, Zhengzhou, Henan, China; 3 Hebei University of Chinese Medicine, Shijiazhuang, Hebei, China; 4 Pediatrics, Xiyuan Hospital of China Academy of Chinese Medical Sciences, Beijing, China

**Keywords:** microglia, neurodevelopmental continuum, neuroinflammation, synaptic pruning, vicious cycle

## Abstract

Rather than viewing classical neurodevelopmental disorders (NDDs) such as autism spectrum disorder (ASD) and severe psychiatric illnesses like schizophrenia (SCZ) as isolated diagnostic silos, modern biological psychiatry increasingly conceptualizes them along a shared neurodevelopmental continuum. Although altered synaptic connectivity and sustained immune dysregulation are both well-documented pathological features, the mechanistic crosstalk between them remains incompletely understood. In this review, we propose that microglia occupy a central position at the interface of these processes because of their dual roles in circuit remodeling and immune surveillance. While individual components, including complement-mediated pruning and inflammasome activation, have been extensively studied, the novelty of our framework lies in integrating these fragmented findings into a cohesive, bidirectional Dysregulated Pruning-Neuroinflammation Cycle to describe how the disruption of microglial homeostasis can sustain a self-perpetuating pathological loop. In this model, the vicious cycle may be initiated through an Outside-In route in which environmental inflammatory insults disrupt synaptic pruning, or through an Inside-Out route in which aberrant synaptic elimination releases danger signals that promote neuroinflammation. By considering early-onset ASD and adolescent/early-adult-onset SCZ, we propose that the interplay among timing, patterning, and background biases neurodevelopmental trajectories toward distinct clinical outcomes. Accordingly, this model highlights disease-modifying therapeutic strategies aimed at restoring microglial homeostasis to potentially mitigate the self-sustaining pathology in these clinically significant neurodevelopmental and psychiatric disorders.

## Graphical Abstract

**Figure:**
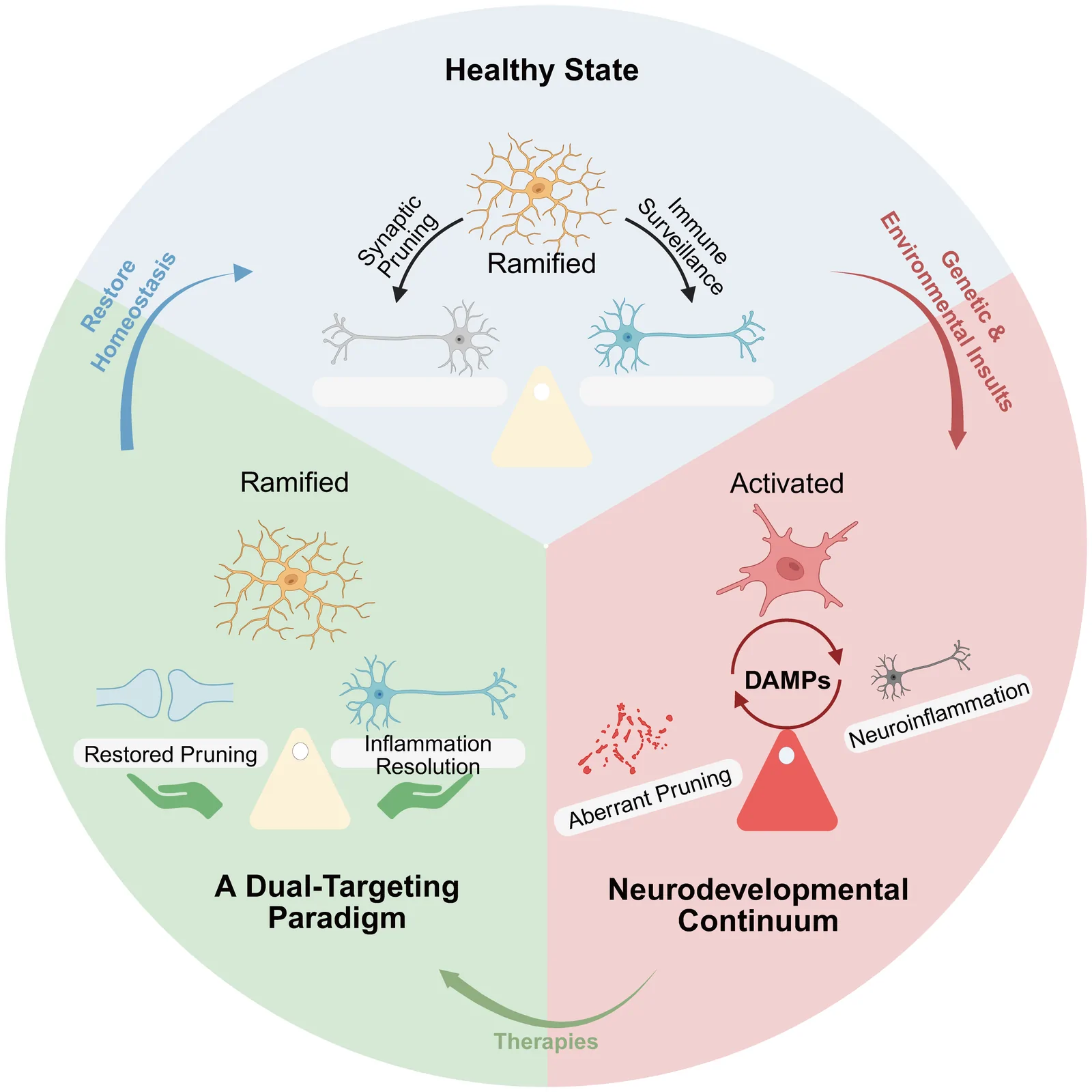
Restoring Microglial Homeostasis Across the Neurodevelopmental Continuum. Schematic overview contrasting the physiological neuroglial state with the pathological vicious cycle, alongside the proposed dual-targeting therapeutic paradigm. (Top) Healthy State: Under homeostatic conditions, ramified microglia maintain a delicate equilibrium between physiological synaptic pruning and continuous immune surveillance, ensuring proper neural circuit refinement. (Bottom Right) Pathological State: Genetic vulnerabilities and environmental insults disrupt this balance. Microglia shift into a primed, activated state, trapping the network in a vicious cycle where aberrant synaptic pruning (either excessive or insufficient) and DAMP-driven neuroinflammation mutually amplify each other, driving the clinical phenotypes observed across the neurodevelopmental continuum. (Bottom Left) A Dual-Targeting Paradigm: To break this self-sustaining loop, emerging therapeutic strategies must adopt a dual-action approach. By simultaneously promoting inflammation resolution and restoring synaptic pruning homeostasis, these interventions aim to reprogram microglia back to an approximate resting state, thereby re-establishing structural and immunological equilibrium within the CNS.

## 1 Introduction

Rather than being viewed as isolated diagnostic silos, classical neurodevelopmental disorders (NDDs) such as autism spectrum disorder (ASD) and severe psychiatric illnesses like schizophrenia (SCZ), are increasingly recognized as interconnected conditions along a shared neurodevelopmental continuum that profoundly burdens global health (1), affecting approximately 0.8% and 0.3% of the global population, respectively (2, 3). Despite their distinct clinical features, these conditions share overlapping genetic vulnerabilities and early-life environmental risks. Current clinical interventions remain largely symptom-oriented. These situations indicate that our understanding of the core pathological mechanisms is still incomplete, which underscores the need for an integrative framework that bridges the major cellular and molecular drivers of disease trajectories.

The pathogenesis of these disorders has traditionally been informed by the connectopathy hypothesis and the neuroimmune hypothesis. While the intersection of these two perspectives is increasingly acknowledged, they are still often discussed separately rather than being combined within a common framework. The connectopathy hypothesis suggests that aberrant synaptic pruning is central to the pathology, proposing that alterations in the pathway of synaptic elimination may influence distinct clinical outcomes (4–6). Within this framework, ASD is commonly associated with reduced pruning and relative hyperconnectivity, whereas SCZ is linked to excessive synaptic elimination and subsequent hypoconnectivity (7, 8). Meanwhile, the neuroimmune hypothesis characterizes the pathology as a chronic low-grade neuroinflammation driven by sustained microglial activation (9, 10). In this process, microglia shift from a homeostatic surveillance phenotype toward a persistent reactive state (11, 12). Despite increasing interest in immune–synapse crosstalk, a deeper and more integrative framework bridging connectopathy and neuroimmune dysregulation is still lacking.

Given their dual roles in synaptic elimination and immune surveillance, microglia are well positioned as a mechanistic cellular node bridging these two pathological hypotheses (13–15). Emerging clustered regularly interspaced short palindromic repeats interference (CRISPRi)-based functional genomics data provide convergent support for this framework, for example, perturbation of the ASD risk gene activity-dependent neuroprotector homeobox (*ADNP*) alters microglial endocytic trafficking and impairs synapse-related uptake/pruning phenotypes (16).

Building on these observations, we propose the Dysregulated Pruning-Neuroinflammation Cycle as a mechanistically integrative model linking connectopathy with neuroimmune dysregulation. The primary novelty of our framework lies in conceptualizing these previously isolated components into a bidirectional, self-reinforcing loop. This cycle is driven by two distinct routes, with an “Outside-In” pathway in which the inflammatory milieu reciprocally distorts microglia-mediated synaptic pruning, and an “Inside-Out” pathway in which aberrant circuit disorganization initiates sterile inflammatory signaling. Within this dynamic framework, we compare early-onset ASD with adolescent/early-adult-onset SCZ to illustrate how the interplay among developmental timing, pruning patterning, and biological background shapes divergent clinical trajectories. Therapeutically, this framework shifts the goal from symptom management to disease modification by breaking self-sustaining pathological cycle and restoring microglial homeostasis. Importantly, although we place microglia at the epicenter of this pathological loop, we emphasize that they do not act in isolation. Rather, they serve as a central coordinating node within a highly integrated, multicellular neuroglial network. Within this dynamic unit, astrocytes actively regulate complement production and blood-brain barrier (BBB) integrity, while neurons are not merely passive targets, but dynamically dictate microglial behavior by presenting specific “eat-me” and “don’t-eat-me” signals.

## 2 Microglia: the dynamic architects and sentinels of neurodevelopment

As previously outlined, the pathogenesis of neurodevelopmentally rooted conditions is consistently associated with two interrelated hallmarks, namely aberrant synaptic connectivity and chronic low-grade immune activation (4–6, 9, 10). Microglia uniquely possess the dual physiological capacity to sculpt synapses and orchestrate neuroinflammation, positioning them as a critical cellular intersection that bridges these seemingly distinct pathologies (13–15). This proposed positioning is consistent with emerging functional genomics data. For instance, CRISPRi-based models show that perturbation of specific neurodevelopmental risk genes, such as *ADNP*, can impair microglial endocytic and synapse-pruning phenotypes (16). Synthesizing these connectomic, neuroimmune, and functional genomic premises, we propose that disorders rooted in early developmental perturbations, ranging from classical NDDs to SCZ, may arise in part from disrupted microglial homeostasis. In this framework, microglia fail to maintain the delicate balance between their dual roles in neural circuit remodeling and immune defense. However, fully appreciating how such pathological dysfunction unfolds requires a clear view of the normal developmental programs and physiological mechanisms that govern microglia in the healthy brain. Accordingly, this section traces the biology of microglia from their embryonic origin and maturation through the molecular machinery underlying synaptic pruning, thereby establishing a mechanistic foundation for understanding how circuit refinement may become perturbed under disease-related conditions.

### 2.1 Origin and development of microglia

Microglia differ from other tissue macrophages in their unique developmental origin. They originate from early erythromyeloid progenitors in the embryonic yolk sac and colonize the developing central nervous system (CNS) independently of definitive hematopoiesis (17, 18). The survival and maintenance of microglia depend on colony-stimulating factor 1 receptor (CSF1R) signaling (19). Furthermore, the differential distribution of the CSF1R ligands CSF-1 and interleukin-34 (IL-34) across different brain regions creates specific conditions for microglial survival, thereby contributing to regional specificity of microglia (20).

As the nervous system develops, microglia shift from motile, amoeboid precursor cells to the highly ramified morphology characteristic of the mature brain (21). This process is regulated by key transcription factors such as PU.1 and IRF8, which also govern the execution of critical microglial functions, including immune surveillance and synaptic remodeling (22). Single-cell transcriptomic studies have shown that even minor disruptions in this developmental trajectory can exert lasting effects on microglial function. This finding aligns with evidence linking early developmental disturbances to an increased risk of neuropsychiatric disorders later in life (23).

### 2.2 Phenotypic and functional diversity of microglia

Microglia show profound phenotypic and functional diversity that varies across different developmental stages and local microenvironments (11). For instance, microglia in the cerebellum typically possess fewer processes, whereas those in the cortex and hippocampus exhibit highly branched and complex structures. These distinct morphologies underscore the remarkable regional specificity of microglia. This regional specificity may reflect varying local demands for synaptic surveillance across different brain regions (24), while simultaneously highlighting the adaptability of microglia to specific functional requirements. Microglia in white matter serve as a prime example, as they initiate transcriptional programs critical for myelin regulation (25).

Traditionally, microglia were classified into a resting state (M0), a pro-inflammatory classical activation state (M1), and an anti-inflammatory alternative activation state (M2). Single-cell transcriptomic studies indicate that microglial phenotypes represent a dynamic continuum shaped by local environmental cues, extending well beyond this simplified M1/M2 dichotomy model (26). This continuum encompasses both homeostatic populations characterized by high expression of purinergic receptor P2Y12 (*P2RY12*) and C-X3-C Motif Chemokine Receptor 1 (*CX3CR1)*, and developmental subpopulations that emerge transiently during specific windows of neural circuit remodeling. Notably, subsets displaying secreted phosphoprotein 1 (*SPP1*)-linked transcriptional features have been implicated in synapse-related phagocytic and pruning programs in specific developmental contexts (27, 28). Under pathological conditions, homeostatic microglia can shift toward disease-associated microglia (DAM)-like profiles, marked by increased triggering receptor expressed on myeloid cells 2 (TREM2) and apolipoprotein E (APOE) expression, alongside metabolic reprogramming. Alternatively, they may adopt an interferon-responsive microglial (IRM) state maintained by persistent immune signaling (29–31). Such transitions may prime microglia, lowering their activation threshold and amplifying their susceptibility to secondary insults. Consistent with this, maternal immune activation (MIA) models show that early immune disturbances prime microglia, leading to heightened neuroinflammatory and behavioral responses to subsequent challenges (32, 33).

### 2.3 The core role of microglia in synaptic network remodeling

In the developing brain, microglia promote neural circuit refinement and mature functional connectivity by executing synaptic pruning (15, 34). Perturbation of this tightly regulated process may impair the clearance of damaged or redundant synaptic debris, potentially triggering the release of damage-associated molecular patterns (DAMPs) and initiating sterile inflammatory cascades that ultimately disrupt local tissue homeostasis.

#### 2.3.1 Dynamic monitoring and synaptic assessment

Microglial surveillance undergoes dynamic evolution across development, transitioning from embryonic directed migration to persistent, dynamic scanning within the postnatal neuropil (35). This behavior relies not only on rapid, localized remodeling of microglial processes but is regulated by extracellular purinergic signaling. Specifically, ATP and ADP drive the chemotactic polarization and targeted migration of microglia via a P2Y12-dependent pathway (27). Upon contacting synaptic elements, microglia integrate molecular signals that reflect synaptic status and neuronal activity, influencing synaptic fate and function (28, 36). Decisions regarding synaptic fate can be seen as a dynamic balance between pro-phagocytic “eat-me” signals such as complement component 1q (C1q) and complement component 3 (C3), and inhibitory “don’t-eat-me” ligand-receptor pathways, including CD47- signal regulatory protein α (SIRPα) and CD200-CD200 receptor (CD200R) signaling (37, 38).

Notably, microglial appraisal of synapses is most prominent during critical developmental windows and transitions to a relatively lower-activity, homeostatic surveillance mode in the mature brain (35). Across the neurodevelopmental continuum, dysregulation of pathways such as C-X3-C Motif Chemokine Ligand 1- CX3CR1 (CX3CL1-CX3CR1) may impair microglial surveillance and synaptic pruning, allowing inefficient synaptic connections to persist and potentially contributing to connectivity abnormalities commonly reported in ASD (39).

#### 2.3.2 Molecular mechanisms of pruning execution

The initial selection of synapses for pruning is fundamentally an activity-dependent process, heavily instructed by the functional state of the neuron. Experimental data, particularly from classic visual system models, demonstrate that less active or weaker synapses are preferentially targeted for elimination, whereas highly active synapses are protected and stabilized (15, 40, 41). Once a synapse is selected for removal, the balance between pro-phagocytic and inhibitory cues determines the mode and extent of pruning. Vulnerable synapses are often tagged through complement-dependent mechanisms, where C1q and C3 deposition facilitates recognition by microglial complement receptor 3 (CR3) (15, 37). An alternative pathway involves externalization of anionic lipids, such as phosphatidylserine (PS), which are sensed by lipid-responsive programs and engage phagocytic signaling, including TREM2-associated signaling (42). Whether a synapse undergoes full engulfment or only partially stripped via microglial trogocytosis is governed by the balance of “eat-me” and “don’t-eat-me” cues (43).

In neurodevelopmentally rooted conditions, disruptions of these signals can perturb synaptic pruning. In SCZ, genetic variations related to complement pathways are linked to altered synaptic tagging and an increased risk of excessive pruning. Conversely, in Rett syndrome, methyl-CpG binding protein 2 (*MECP2*)-related pathology has been linked to abnormal microglia-synapse interactions and circuit dysfunction (44, 45). Collectively, these findings suggest that tightly regulated molecular mechanisms governing synaptic recognition and clearance are required for precise circuit remodeling.

### 2.4 Microglia as spatiotemporal regulators of neural circuit formation

Microglial regulation of neural circuit assembly is temporally organized and integral to brain maturation. Their functions are spatiotemporally specific across developmental stages. During embryogenesis, microglia contribute to axon tract development and early circuit patterning, whereas they mediate experience-dependent synaptic remodeling in the postnatal brain (40, 46, 47). Importantly, microglial functions are intrinsically embedded within a dynamic and multicellular neuroglial unit, rather than operating in isolation. Astrocytes are indispensable partners in this network, as they regulate complement production, including C3 synthesis for synaptic tagging, provide structural and metabolic support for synapses, and help maintain BBB integrity in ways that shape CNS cytokine signaling (48, 49). Neurons also act as active participants by directing microglial phagocytic behavior through the presentation of critical “eat-me” signals, such as externalized phosphatidylserine and ATP release, as well as protective “don’t-eat-me” signals, including CD47 and CX3CL1. In addition, interactions between microglia and oligodendroglial lineage cells are important for myelination-associated metabolic homeostasis and white matter integrity (15). Therefore, dysregulation along the neurodevelopmental continuum is better interpreted as a disturbance of the broader neuroglial network, within which microglia may serve as a key hub rather than the sole pathological driver.

Under these pathological conditions, neuroglial network coordination may be compromised, yet microglial signaling may still produce region- and circuit-specific effects. For example, in CX3CR1-deficient models, certain neuronal pruning programs in the cerebellum remain preserved, indicating that microglial signaling dependencies vary heavily across neural circuits (50). Conversely, in circuits strictly reliant on these specific pathways, localized dysfunction can shift microglia from homeostatic regulators to primary contributors to aberrant connectivity.

## 3 Microglia-mediated neuroinflammatory signaling: specificity in the developmental context

Microglia bridge neural circuit remodeling and innate immune defense, two major functional axes in the brain. The former supports circuit refinement, whereas the latter can be rapidly activated by genetic vulnerability or environmental perturbations, thereby initiating neuroinflammatory signaling. In the mature brain, such immune responses often exert a double-edged effect. However, in the developing CNS, this balance is disproportionately shifted toward harm, as immune programs recruited to counter pathological stimuli may compromise the integrity of immature neural circuits. This section outlines the signaling pathways that initiate and amplify microglia-mediated neuroinflammation, and further discusses how defects in inflammation-resolution mechanisms may fuel the persistent dysfunction observed across the neurodevelopmental continuum.

### 3.1 Neuroinflammation during development: a sharper “double-edged sword”

Neuroinflammation is typically characterized by microglial activation and release of soluble inflammatory mediators that can induce local tissue injury, with peripheral immune-cell recruitment occurring particularly when BBB integrity is compromised or inflammation is sustained (51). In the mature CNS, inflammatory responses can serve as important protective and reparative mechanisms, however, when persistent or dysregulated, they carry a substantial risk of collateral damage (52). This risk is further amplified in the developing brain, a critical window during which active neuronal migration, circuit assembly, and myelination render the CNS especially vulnerable. As a result, even transient inflammatory insults may produce lasting structural and functional impairments (53, 54).

Microglial priming is a key contributor to this heightened developmental vulnerability. Early-life stressors such as MIA and neonatal hypoxia, may not cause obvious, widespread neuroinflammation at the time of exposure, but they can induce a persistent sensitized microglial state (54, 55). Primed microglia exhibit a lowered threshold for reactivation and generate exaggerated responses to subsequent, otherwise mild, stimuli (56). Preclinical models further show increased cytokine release following a second hit, suggesting that priming establishes an innate immune memory-like program (57). In turn, such sensitization may convert protective immune surveillance into chronic neuroinflammatory dysregulation, thereby disrupting critical developmental processes such as synaptic pruning.

### 3.2 The activated microglial phenotype: from cellular remodeling to effector functions

During activation, microglia undergo both morphological and metabolic remodeling. Their highly ramified surveillance processes retract, and the cells shift toward a highly motile, amoeboid phenotype. In parallel, microglia undergo metabolic reprogramming, typically showing increased glycolytic flux with a relative reduction in oxidative phosphorylation, to meet the surging bioenergetic and biosynthetic demands of inflammatory responses (58–60). Such metabolic reprogramming not only provides the ATP and anabolic intermediates required for phagocytosis but also supports upregulation of antigen presentation pathways of major histocompatibility complex class II (MHC-II) in specific inflammatory contexts (61).

The inflammatory response is initiated when pattern recognition receptors (PRRs) sense danger cues and trigger signaling cascades that activate transcriptional programs such as nuclear factor-κB (NF-κB), thereby upregulating pro-inflammatory gene expression (62, 63). The stability and translation of these transcripts are further regulated by mitogen-activated protein kinase (MAPK) cascades, especially p38 and c-Jun N-terminal kinase (JNK), which also influence stress-related transcriptional responses. Meanwhile, the janus kinase/signal transducer and activator of transcription (JAK/STAT) pathway integrates extracellular cytokine signals to shape microglial phenotypes (64–66). Under physiological conditions, these pathways are constrained by intrinsic counter-mechanisms. Notably, the phosphoinositide 3-kinase/protein kinase B (PI3K/Akt) axis can dampen excessive pro-inflammatory signaling to limit overactivation in specific contexts (67, 68).

Activated microglia release a broad repertoire of effector molecules that not only reshape the local milieu but also impair the neurovascular unit. Pro-inflammatory cytokines, including tumor necrosis factor-α (TNF-α) and interleukin-1 β (IL-1β), can compromise synaptic plasticity partly by suppressing long-term potentiation (LTP), wherein IL-1β bioactivity is regulated in part by NLRP3 inflammasome-dependent processing (61, 69). Concurrently, these inflammatory mediators downregulate tight junction proteins, increase vascular permeability, and weaken BBB integrity (70). This permits chemokines such as C-C Motif Chemokine Ligand 2 (CCL2) and C-X-C Motif Chemokine Ligand 10 (CXCL10) to establish chemotactic gradients that promote recruitment of circulating monocytes and T cells into the CNS parenchyma, thereby attenuating immune privilege of the CNS (71, 72). Interactions between resident microglia and infiltrating peripheral immune cells then sustain and amplify inflammatory signaling, accompanied by increased production of neurotoxic reactive oxygen species (ROS) and prostaglandins, which further exacerbates oxidative stress within developing neural circuits (73, 74).

### 3.3 Disrupting the blueprint: how inflammatory mediators impair neurodevelopment

During neurodevelopment, inflammatory mediators can disrupt molecular programs required for synaptic stabilization and maintenance, thereby perturbing circuit maturation. This interference primarily manifests as an abnormal upregulation of the pro-phagocytic pathway. Cytokines released during neuroinflammation (such as TNF-α and IL-1β) can increase the expression of complement components C3 and C1q in neurons and astrocytes (75, 76). Excessive synaptic tagging hyperactivates CR3-dependent microglial pruning, leading to pathological synapse loss, a phenomenon observed in SCZ models (77). Furthermore, these mediators may also impair functional connectivity. For example, TNF-α can weaken synaptic strength and impair synaptic function by promoting the aberrant endocytosis of AMPA receptors (78).

More broadly, the inflammatory milieu can disrupt developmental processes beyond synapses, including suppressing neural stem cell (NSC) proliferation and skewing cell differentiation from neuronal differentiation (69). Oligodendrocyte development is similarly impacted, contributing to hypomyelination and white-matter abnormalities observed in NDDs (70, 79). Overall, these results suggest that neuroinflammation is not merely a secondary consequence within the neurodevelopmental continuum, but rather act as a major catalyst in the process of circuit dysregulation.

### 3.4 Resolution of neuroinflammation: from negative feedback to active repair

Resolution of neuroinflammation is not merely a passive decay of inflammatory signaling, but an active and tightly controlled program. Intracellularly, this process involves multiple counter-regulatory mechanisms, including inducible IκBα expression that restrains NF-κB activity and the upregulation of suppressor of cytokine signaling (SOCS) family members that suppress JAK/STAT signaling, together dampening pro-inflammatory transcriptional programs (62).

At the tissue level, inflammation resolution depends heavily on anti-inflammatory signals that remodel the extracellular milieu, notably interleukin-10 (IL-10) and transforming growth factor-β (TGF-β). These mediators help constrain excessive glial activation and support restoration of BBB function (80–82). Critically the inhibitory, pro-repair microenvironment shaped by anti-inflammatory cytokines creates conditions favorable for neuronal recovery and helps re-engage homeostatic signaling axes such as CX3CL1-CX3CR1, thereby facilitating restoration of microglial surveillance functions (83).

Furthermore, inflammatory resolution depends on the biosynthesis of specialized pro-resolving mediators (SPMs), including resolvins and protectins. These bioactive lipids dampen immune responses by limiting further leukocyte infiltration and accelerating clearance of apoptotic cells and tissue debris (84–86). Emerging evidence suggests that pro-resolving pathways may be functionally impaired in at least a subset of neurodevelopmental conditions (85).

## 4 Microglial dysfunction: the convergence of aberrant pruning and neuroinflammation

Building on current understanding of microglial biology, and strongly supported by the aforementioned genetic susceptibilities (e.g., genome-wide association studies and CRISPRi data) and clinical neuropathological premises, we propose that the long-term impact of these neurodevelopmentally rooted conditions arises from dysregulation of the mechanisms that normally balance microglial functions. We hypothesize that, across a subset of this continuum, diseases such as ASD and SCZ are significantly propelled by a failure of microglia to maintain equilibrium between synaptic pruning and immune surveillance. Under pathological conditions, these two programs become maladaptively coupled, generating reciprocal crosstalk between aberrant pruning and neuroinflammation that traps brain networks in a self-sustaining state of chronic dysfunction. In this view, the Dysregulated Pruning–Neuroinflammation Cycle may provide a mechanistic framework for understanding how transient developmental insults can be converted into long-term, circuit-level deficits.

### 4.1 Entry points to microglial dysfunction: pruning failure and neuroinflammatory bias

Synaptic pruning may fail from defects across different stages of microglial function, including environmental surveillance, signal integration, and phagocytic execution. These impairments may be precipitated by genetic susceptibility and environmental stress, fundamentally reshaping how microglia sense, evaluate, and remodel developing circuits.

A common early alteration is the loss of effective microglial immune surveillance. Under homeostatic conditions, microglia continuously patrol neural tissue via highly dynamic processes. However, evidence from ASD- and SCZ-related studies indicates that disruption of the CX3CR1 signaling axis has been linked to reduced process motility and may create a timing mismatch in developmental pruning, thereby increasing the risk of missing critical pruning windows (87, 88). Immune surveillance is similarly vulnerable to environmental perturbation. Such perturbations can shift fetal microglia toward a low-dynamic amoeboid phenotype, constraining spatial coverage and fine-scale monitoring, and promoting less selective response patterns (87).

Erroneous synaptic tagging further disrupts the balance between “eat me” and “don’t eat me” signals by enhancing pro-phagocytic signals and attenuating protective inhibitory signals, thereby exacerbating synaptic elimination. This dysregulation primarily stems from complement cascade abnormalities and exhibits disease specificity. Specifically, in SCZ, genetic risk variants can upregulate Complement component 4 (*C4*) expression, leading to excessive synaptic tagging and subsequent aberrant elimination (8, 44). In certain ASD models, however, abnormal C1q deposition can drive microglia to eliminate otherwise healthy synaptic connections (89, 90). Moreover, functional insufficiency of protective signaling molecules (such as CD47) may impair the ability to retain functional synapses, thereby exacerbating this pathological clearance (91).

These early-stage functional abnormalities ultimately lead to aberrant synaptic pruning. Across the neurodevelopmental continuum, microglia often reside in a primed state, characterized by a lowered activation threshold and dysregulated phagocytic activity. This state likely serves as a major contributing factor underlying the excessive synaptic elimination observed in SCZ (92, 93). In contrast, microglia in Rett syndrome models exhibit diminished phagocytic capacity, resulting in inefficient clearance of cellular debris and the persistence of redundant synaptic connections (94). These divergent phagocytic behaviors, ranging from excessive to insufficient clearance, illustrate how dysfunction at any stage of microglial activity can precipitate aberrant patterns of synaptic connectivity.

In addition, when immune responses become dysregulated and inflammation fails to resolve, microglia lose their homeostatic state, precipitating severe cellular dysfunction (81, 86). PRRs are critical to this process, as they convert endogenous danger signals and inflammatory exposures into cytokine release and inflammasome activation. This cascade of events disrupts synaptic tagging and clearance mechanisms (62, 76, 95, 96). Failure to engage pro-resolving pathways and restore homeostasis results in sustained neuroinflammation, locking microglia into a chronic reactive state (55, 81, 84, 86). Early-life insults, such as MIA or hypoxia, lower microglial activation thresholds, priming them to become hyper-responsive to subsequent inflammatory stimuli (33, 55–57). Taken together, these findings suggest that multiple etiological risks converge on a central node of microglial dysfunction, where synergistic interactions between disrupted pruning and inflammation may contribute to the shared pathology (14, 97).

### 4.2 The pathogenic loop: integrating synaptic errors with neuroinflammation

The foundational concept that microglial dysfunction and immune activation contribute to neurodevelopmental and psychiatric disorders has been established by pioneering research over the past decades (56, 98). Building upon these crucial contributions, we seek to formalize and integrate these well-established phenomena into a cohesive, bidirectional framework. To understand how these developmental aberrations progress into chronic pathologies, we propose a model that bridges synaptic remodeling and neuroimmune activation. We posit that this relationship is not linear but rather a bidirectional feedback loop triggered by an interplay of genetic and environmental factors. By categorizing these interactions into “Inside-Out” and “Outside-In” routes, we illustrate how these previously described biological mechanisms operate not in isolation, but as a self-sustaining vicious cycle.

The Inside-Out pathway conceptualizes how initial defects in synaptic elimination can trigger secondary neuroinflammation. Unlike the silent, non-inflammatory clearance of synapses under physiological conditions, excessive pruning or inefficient clearance causes the accumulation of synaptic debris. This exacerbates local cellular stress, resulting in the release of DAMPs, such as ATP and mitochondrial DNA (mtDNA) (99). These danger signals are recognized by microglial PRRs, thereby initiating a sterile inflammatory response. Specifically, mtDNA engages TLR9, while ATP activates the NLRP3 inflammasome via the P2X7 receptor signaling (95, 96). Although direct *in vivo* evidence demonstrating that pruning defects strictly precede and drive inflammasome activation in models of ASD and SCZ remains limited, converging mechanistic and indirect experimental data support the plausibility of this sequence. Within this framework, circuit-level dysfunction may act as an upstream trigger of aberrant immune activation, which can not only persist but may even surpass the initial synaptic pruning deficit in severity.

The Outside-In pathway delineates how a primary inflammatory environment compromises synaptic integrity. Drawing from extensively characterized MIA models, this cascade exemplifies how a single prenatal inflammatory event is sufficient to disrupt microglial maturation, thereby consolidating a persistent hyperresponsive phenotype (33, 98). Within this altered milieu, genetic susceptibility factors, such as *C4* variants, act as amplifiers, increasing pathological synaptic tagging while rendering synaptic connections highly vulnerable to subsequent inflammatory insults (8, 44, 100).

**Figure 1:**
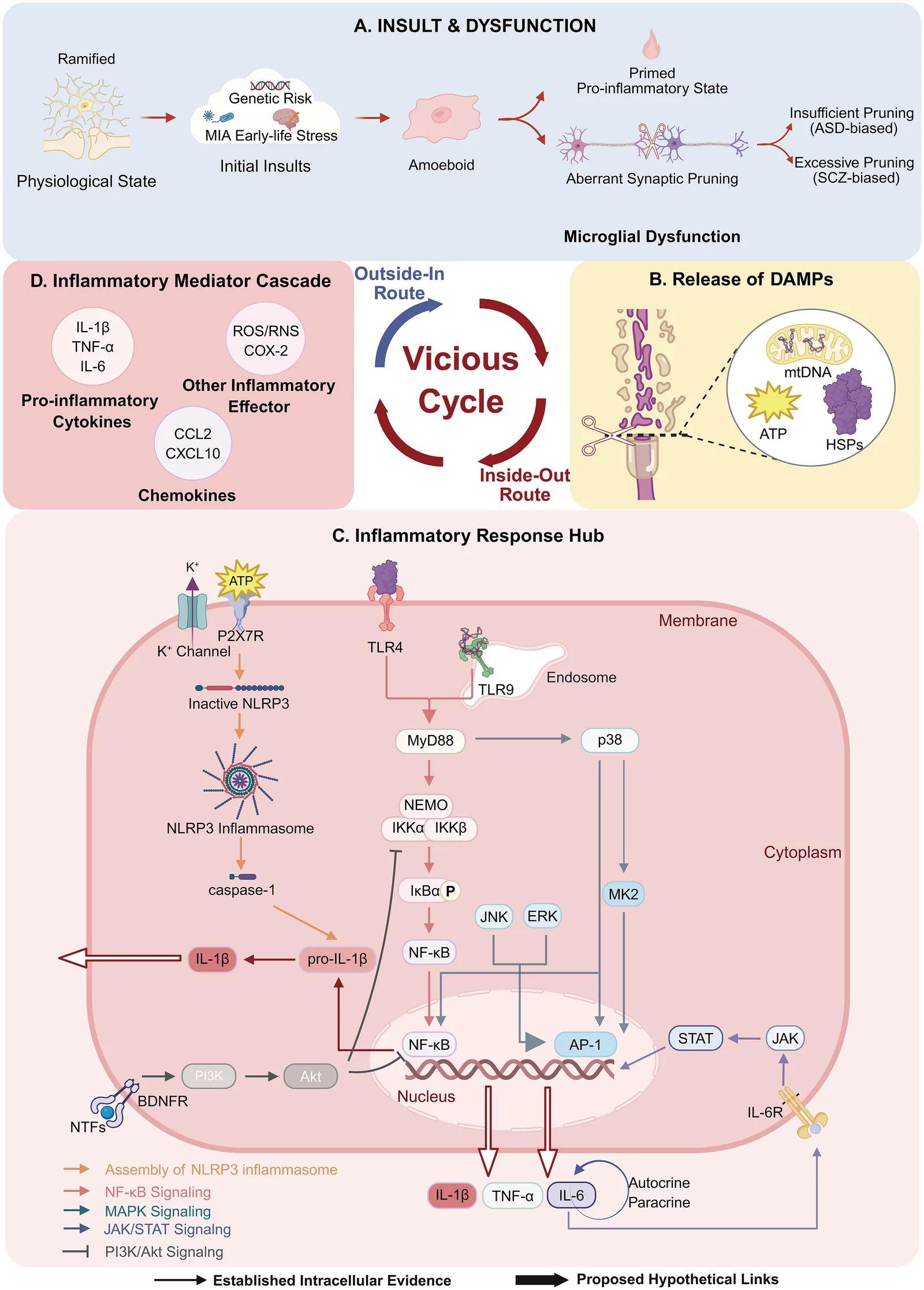
The “Dysregulated pruning-neuroinflammation” vicious cycle. Schematic illustrating the self-sustaining feedback loop that exacerbates neuroinflammation and synaptic pathology across the neurodevelopmental continuum. **(A)** Insult & Dysfunction: Genetic vulnerabilities or environmental stressors initiate microglial dysfunction, shifting cells toward a primed pro-inflammatory state while simultaneously disrupting synaptic pruning. The aberrant synaptic pruning bifurcates into either insufficient pruning (ASD-biased trajectories) or excessive pruning (SCZ-biased trajectories) depending on the specific developmental context. **(B)** Release of DAMPs: This pruning failure liberates Damage-Associated Molecular Patterns (DAMPs), including mtDNA and ATP, from damaged synaptic structures. **(C)** Inflammatory Response Hub: Microglial pattern recognition receptors (e.g., TLRs, P2X7R) sense these DAMPs to trigger intracellular signaling cascades, specifically activating NF-κB and assembling the NLRP3 inflammasome. Together, A→B→C delineates the proposed “Inside-Out” route. **(D)** Inflammatory Mediator Cascade: The release of pro-inflammatory cytokines (IL-1β, TNF-α) and chemokines creates a neurotoxic milieu. As depicted by the “Outside-In” route (D→A), this primary inflammatory environment feeds back to disrupt microglial maturation and further exacerbate the initial pruning defects, effectively locking the neuroglial network in a dysregulated loop. Akt, Protein Kinase B; AP-1, Activator protein 1; ATP, Adenosine triphosphate; BDNFR, Brain-derived neurotrophic factor receptor; CCL2, C-C motif chemokine ligand 2; COX-2, Cyclooxygenase-2; CXCL10, C-X-C motif chemokine ligand 10; DAMPs, Damage-associated molecular patterns; ERK, Extracellular signal-regulated kinase; HSPs, Heat shock proteins; IKKα/β, IκB kinase alpha/beta; IκBα, Inhibitor of kappa B alpha; IL-1β, Interleukin-1 beta; IL-6, Interleukin-6; IL-6R, Interleukin-6 receptor; JAK, Janus kinase; JNK, c-Jun N-terminal kinase; MIA, Maternal immune activation; MK2, MAPK-activated protein kinase 2; mtDNA, Mitochondrial DNA; MyD88, Myeloid differentiation primary response 88; NDDs, Neurodevelopmental disorders; NEMO, NF-κB essential modulator; NF-κB, Nuclear Factor-kappa B; NLRP3, NOD-like receptor protein 3; NTFs, Neurotrophic factors; p38, p38 mitogen-activated protein kinase; P2X7R, P2X7 purinergic receptor; ROS/RNS, Reactive oxygen/nitrogen species; STAT, Signal transducer and activator of transcription; TLRs, Toll-like receptors; TNF-α, Tumor necrosis factor-alpha.

Although these pathways may initially appear independent, they ultimately converge to establish a self-sustaining state of cellular dysfunction. Once this cycle is engaged, within this framework neuroinflammation becomes a key amplifier of disease progression. Cytokines like TNF-α and IL-1β, for instance, not only directly impair synaptic function but also promote aberrant complement deposition. This engenders a vicious cycle in which hyperactivated microglia accelerate synaptic clearance and release additional danger signals, thereby further amplifying the inflammatory response (14). Such reciprocal interactions may explain how even transient or mild developmental insults can precipitate enduring deficits in neural circuit function (**Figure 1**).

## 5 The vicious cycle in action: pathological evidence across the neurodevelopmental continuum

Drawing from both clinical and preclinical studies, we recognize that multiple etiological factors ultimately converge on a central pathological mechanism centered on microglial dysfunction. The reciprocal amplification between synaptic impairment and immune dysregulation may contribute to the pathogenesis of neurodevelopmentally rooted conditions. Rather than operating as parallel processes, these mechanisms are deeply intertwined and inseparable throughout development. We therefore propose a pathological model of a Dysregulated Pruning–Neuroinflammation Cycle. Evidence from ASD and SCZ, encompassing genetic risk factors, functional genomics findings, and postmortem neuropathological observations, provides foundational support for this unified framework. Building upon this foundation, we further appreciate that specific disease phenotypes arise from dynamic interactions among three cardinal elements, namely Timing, Patterning, and Background. These interactions constitute a fundamental basis for the clinical heterogeneity observed across the neurodevelopmental continuum.

### 5.1 ASD: a failure of refinement amidst chronic immune vigilance

The neuropathology of ASD exhibits aberrant neural circuit organization, often accompanied by persistent neuroimmune activation, which supports the vicious cycle model proposed here. Autopsy studies have revealed increased microglial density in key brain regions, including the prefrontal cortex, with reactive microglial morphologies predominating (101, 102). These findings do not point to a single, uniform lesion but rather indicate that, at least in some ASD patients, persistent alterations in the glial homeostasis mechanisms responsible for circuit refinement initiate this pathological cycle.

Genomic evidence further substantiates the immune-synaptic vulnerability concept, with high-confidence ASD risk genes significantly enriched in pathways related to synaptic function and immune regulation (103, 104). Functional genomics is now beginning to link these genetic risks to alterations in microglial activity. In CRISPRi-based screens, knockdown of ASD-associated genes, such as *ADNP*, impairs microglial phagocytic function, thereby linking genetic susceptibility to altered synaptic pruning phenotypes across different ASD subtypes (16). Concurrently, clinical studies report elevated pro-inflammatory cytokine levels in the cerebrospinal fluid of ASD patients, suggesting sustained immune dysfunction in at least a subset of cases (70, 97, 102).

Animal models have directly provided strong evidence that inflammatory exposure can alter the normal developmental trajectory of microglia. In MIA models, a single prenatal inflammatory insult is sufficient to induce sustained microglial activation, accompanied by lasting changes in synaptic pruning and behavioral phenotypes (105, 106). Furthermore, minocycline treatment and interleukin-17A (IL-17A) pathway blockade can alleviate ASD-like phenotypes in MIA offspring, providing evidence that immune dysregulation and microglia-mediated neuroinflammation are potential pathogenic contributors in ASD (107, 108). However, it is imperative to acknowledge that ASD is a highly heterogeneous spectrum rather than a monolithic disorder. Despite the predominant features of synaptic refinement defects in ASD, certain genetic models exhibit an opposing pattern. In these cases, microglia are involved in excessive synapse elimination, suggesting that dysregulated microglial function may result in different synaptic outcomes depending on the underlying molecular context (89).

### 5.2 SCZ: an adolescent cascade of synaptic over-pruning

Unlike the early onset of ASD, SCZ typically emerges during adolescence, coinciding with the later stages of cortical synaptic refinement. This timing aligns with genetic susceptibility, which often remains clinically silent until synapses undergo a more substantial remodeling. Genomic studies have largely identified structural variations at the complement *C4* locus, with risk alleles linked to increased expression of *C4A*. This leads to enhanced complement-dependent synaptic tagging, promoting microglial clearance of synapses (44, 109).

Complement-related risk provides a molecular entry point for the maladaptive cycle we propose, with its effects being most prominent during the plasticity phase in adolescence. In a mouse model, *C4A* overexpression is largely normal during early life, but later in adolescence, it leads to increased synaptic C3 deposition and enhanced microglial phagocytic activity. These changes correlate with reduced dendritic spine density in the prefrontal cortex and the onset of cognitive and social deficits (8). Clinical evidence also supports enhanced microglia-mediated synaptic clearance in cell systems associated with SCZ (77).

Although obtaining longitudinal evidence of synaptic pruning at the level of individual synapses remains challenging in clinical studies, neuroimaging and autopsy data both suggest an accelerated loss of cortical connectivity. Neuroimaging studies report an accelerated loss of cortical gray matter in SCZ patients (110, 111). Autopsy analysis corroborated these findings, recording a 20-30% reduction in dendritic spine density of pyramidal neurons in the corresponding cortical areas (112). Collectively, these observations support a synaptic pruning mechanism in which excessive cortical remodeling during adolescence leads to synaptic elimination, driving the onset and progression of SCZ. Nevertheless, SCZ is a multifactorial psychiatric illness. Although complement-mediated over-pruning represents a major pathogenic cascade, it is likely to interact synergistically with other established mechanisms, including neurotransmitter imbalances and interneuron dysfunction, to shape the full clinical phenotype (113, 114).

### 5.3 A spectrum of dysfunction: the interplay of timing, patterning, and background

As previously discussed, we propose that although ASD and SCZ are both associated with microglial dysfunction, significant differences between them suggest that the pathology of this spectrum is not a single event, but rather a continuum influenced by Timing, Patterning, and Background. We propose that the interaction of these three key factors with the pathological vicious cycle ultimately determines the specific clinical manifestations (**Table 1**).

**Table 1:** Operational definitions of timing, patterning, and background in the dysregulated pruning–neuroinflammation cycle across ASD and SCZ.

Dimension	Operational definition	Proposed operation in ASD	Proposed operation in SCZ	Interpretive caution
Timing	The developmental window during which neuroimmune perturbation, microglial priming, or synaptic remodeling abnormalities occur. Timing determines which circuits are vulnerable when the pruning–inflammation cycle is engaged.	Early embryonic development/Early postnatal stages (33, 105, 106)	Adolescence/Early adulthood (8)	Timing should not be interpreted as a rigid developmental boundary. ASD- and SCZ-related processes may overlap across developmental stages, and early-life perturbations may influence later adolescent vulnerability.
Patterning	Patterning denotes the directionality (e.g., excessive versus insufficient), anatomical distribution, and precise molecular mechanisms of synaptic remodeling, which collectively define the final structural architecture of neural networks.	Predominantly insufficient or mistimed pruning (though highly heterogeneous) (53, 89, 115)	Predominantly runaway/excessive microglial pruning (8, 77, 110–112, 116)	ASD should not be reduced to under-pruning, and SCZ should not be reduced to over-pruning. Patterning is likely region-specific, cell-type-specific, developmentally dynamic, and dependent on molecular context.
Background	The biological and environmental substrate that shapes microglial state, inflammatory threshold, and synaptic vulnerability. Background includes genetic risk, biological sex, immune history, microbiome composition, and environmental exposure.	ASD-related background may include high-confidence synaptic and immune risk genes (16, 103, 104) male-biased neuroimmune vulnerability (117, 118), early immune priming, altered cytokine profiles (70, 97, 102), and gut–brain axis dysregulation (119, 120). These factors may bias microglia toward altered surveillance and abnormal circuit refinement during early development.	SCZ-related background may include complement C4A-associated genetic liability (8, 44, 109), immune priming, adolescent second-hit vulnerability, neurotransmitter imbalance and interneuron dysfunction (113, 114). These factors may interact with late cortical pruning demands to promote SCZ-like trajectories.	Background factors are not deterministic. They likely modify risk probabilistically and interact with timing and patterning rather than independently determining diagnostic outcome.
Integrated effect	The combined interaction of timing, patterning, and background determines how a shared microglial pruning–inflammation loop is expressed as distinct or overlapping clinical trajectories.	Early developmental vulnerability combined with altered pruning patterning and ASD-related biological background may favor early-onset social, sensory, and cognitive phenotypes.	Adolescent vulnerability combined with excessive pruning patterning and SCZ-related biological background may favor later-emerging cognitive, negative, and psychotic-spectrum phenotypes.	The model should be viewed as a mechanistic framework rather than a diagnostic classifier. ASD and SCZ may represent partially overlapping outcomes of disrupted immune–synaptic regulation rather than entirely separate biological categories.

ASD, autism spectrum disorder; C4A, complement component 4A; SCZ, schizophrenia.

In the early stages of this cycle, timing refers to the specific developmental windows during which neuroimmune insults occur, determining precisely which neural circuits are rendered vulnerable. Damage occurring during early embryonic development disrupts the formation of basic sensory and social processing systems, leading to the early onset of ASD with multi-domain functional impairments (33). In contrast, pathological impacts occurring in late adolescence selectively target the higher-order associative cortex, which is in the final stages of refinement, providing a mechanistic explanation for the later onset of executive function deficits in SCZ (8). Therefore, the timing of these processes is crucial to understanding why different neural circuits are affected in distinct ways during various developmental stages.

Similarly, patterning denotes the directionality (e.g., excessive versus insufficient), anatomical distribution, and precise molecular mechanisms of synaptic remodeling, which collectively define the final structural architecture of neural networks. In many ASD subtypes, insufficient synaptic pruning generates excessive network connectivity, which severely degrades signal-to-noise ratios. This aberrant architecture provides a physiological basis for the sensory hypersensitivity and sensory overload characteristic of the disorder (53, 115). Conversely, in canonical SCZ, excessive synaptic elimination leads to sparse and inefficient neural networks, thereby promoting the hallmark negative symptoms and severe cognitive decline (116). However, framing ASD exclusively as a disorder of under-pruning and SCZ exclusively as over-pruning represents an oversimplified, binary dichotomy. Given their shared genetic vulnerabilities and overlapping neuroimmune pathways, ASD and SCZ should instead be viewed as heterogeneous and partially overlapping outcomes of disrupted immune-synaptic regulation. Based on the vicious cycle model proposed above, in which synaptic errors and immune activation are mechanistically coupled, ASD and SCZ may share a core microglial pathology characterized by impaired homeostatic surveillance and a chronic shift toward reactive, primed states. Consequently, the affected neuroglial networks may become trapped in a shared pathological loop, which helps explain their overlapping clinical features, including cognitive and social deficits. The ultimate diagnostic trajectory, whether ASD, SCZ, or a comorbid presentation, may therefore be determined not by entirely divergent mechanisms, but by how shared microglial dysfunction intersects spatiotemporally with specific developmental windows and regional circuit vulnerabilities.

Finally, the ultimate clinical outcome is shaped by the individual’s biological background, including genetic risk profile, biological sex, immune history, microbiome composition, and environmental exposures, which together provide the physiological substrate upon which these pathological cycles unfold. This relationship is not linear but arises from the interplay of multiple factors. For instance, prenatal immune exposures combined with a genetic background enriched in complement-related risk alleles strongly biases the outcome toward a SCZ-like phenotype (97). Biological sex also plays a crucial role, as sex differences in microglial development and function offer important clues for the higher prevalence of ASD in males (117, 118). Furthermore, emerging systemic modulators such as the gut microbiome contribute significantly to phenotypic heterogeneity. Microbial metabolites like short-chain fatty acids can actively cross the BBB to shape microglial priming via the gut-brain axis (119, 120).

This integrative framework not only provides a unified model of microglial dysfunction, but also accounts for the substantial clinical variability observed across the neurodevelopmental continuum. Importantly, it may offer insights into personalized therapeutic strategies tailored to individual spatiotemporal and biological contexts.

## 6 Discussion and outlook: targeting microglia across the neurodevelopmental continuum

Recognizing the Dysregulated Pruning-Neuroinflammation Cycle as a key pathology along the neurodevelopmental continuum offers a new therapeutic perspective, namely that clinical intervention should extend beyond symptom-based diagnostic categories and instead target underlying cellular and molecular mediators. By placing microglia at the center of this self-sustaining pathological cycle, our model highlights an opportunity to address disease-specific mechanisms rather than merely manage symptoms, an approach that holds particular value within distinct developmental stages and biological contexts.

### 6.1 Interrupting the cycle: mechanism-based therapeutic strategies

**Figure 2:**
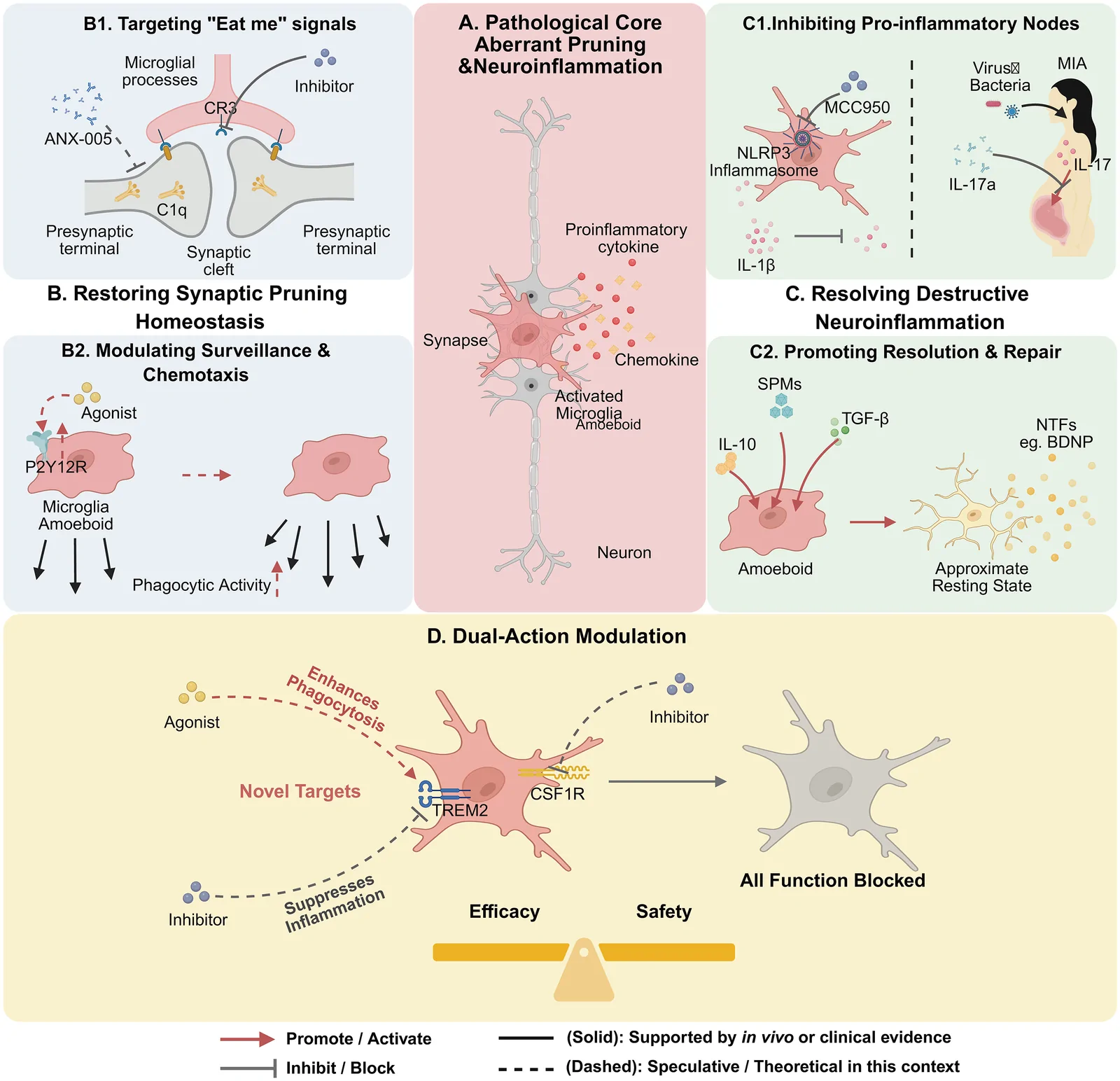
Targeting the microglial axis: a new therapeutic paradigm across the neurodevelopmental continuum. Overview of therapeutic strategies engineered to interrupt the vicious cycle by modulating dual microglial functions. **(A)** Pathological Core: A central dysfunctional microglial cell acts as a key mediating node for both aberrant synaptic pruning and chronic neuroinflammation. **(B)** Restoring Synaptic Pruning Homeostasis: Interventions focus on neutralizing aberrant “eat-me” signals (B1; e.g., via ANX-005, primarily speculative in neurodevelopment) or recalibrating surveillance and phagocytosis (B2; e.g., via P2Y12R agonists) **(C)** Resolving Destructive Neuroinflammation: Strategies include the direct inhibition of pro-inflammatory hubs (C1; e.g., MCC950 targeting the NLRP3 inflammasome, supported by *in vivo* models) or the active promotion of resolution and repair pathways (C2; e.g., SPMs and IL-10). **(D)** Dual-Action Modulation: This advanced approach aims to holistically reprogram microglia (e.g., via TREM2 modulation) to normalize both pathological arms simultaneously. The scale represents the critical need to balance therapeutic efficacy with the preservation of essential physiological functions across distinct development stages. BDNF, Brain-derived neurotrophic factor; C1q, Complement component 1q; CR3, Complement receptor 3; CSF1R, Colony-stimulating factor 1 receptor; IL-10, Interleukin-10; IL-17, Interleukin-17; IL-1β, Interleukin-1 beta; MCC950, A selective NLRP3 inhibitor; MIA, Maternal immune activation; NDDs, Neurodevelopmental disorders; NLRP3, NOD-like receptor protein 3; NTFs, Neurotrophic factors; P2Y12R, P2Y12 purinergic receptor; SPMs, Specialized pro-resolving mediators; TGF-β, Transforming growth factor-beta; TREM2, Triggering receptor expressed on myeloid cells 2.

To break this self-sustaining loop, we propose that therapeutic approaches should target the dual dysfunction of microglia. Our proposed approach is mechanism-driven, aiming to restore proper synaptic elimination by microglia while also resolving their ongoing inflammatory state. And the ultimate goal of these interventions is to restore balance by normalizing both synaptic remodeling and immune function, thereby re-establishing homeostasis in the CNS (**Figure 2**).

#### 6.1.1 Re-calibrating the architect: restoring synaptic pruning homeostasis

One potential strategy to restore circuit connectivity involves directly targeting the molecular pathways that govern complement-dependent synaptic tagging and elimination. Complement inhibition has been suggested as a promising approach to limit excessive synapse elimination in conditions such as SCZ (121, 122). Anti-C1q antibodies such as ANX-005 have been shown to inhibit the initiation of the classical complement cascade (123), thereby reducing downstream complement deposition and mitigating synaptic damage. However, it is crucial to acknowledge that the current evidence supporting ANX-005 and similar agents is primarily derived from autoimmune and neurodegenerative disease contexts. Their therapeutic efficacy in SCZ remains unclear, particularly in patients whose pathology is driven by non-complement mechanisms such as neurotransmitter imbalances. Furthermore, their broader applicability across the neurodevelopmental continuum is highly speculative. Furthermore, administering such anti-complement therapies in ASD subtypes characterized by insufficient pruning could paradoxically exacerbate hyperconnectivity and worsen clinical symptoms.

Another promising approach is to target phagocytosis-related receptors, such as TREM2 and P2Y12R, to modulate microglial engulfment and reactivity. Studies have shown that TREM2 plays an essential role in microglia-mediated synaptic elimination and circuit refinement (124–126). This suggests that enhancing or restoring TREM2-linked signaling may help correct phenotypic abnormalities arising from insufficient synaptic elimination, such as those observed in certain ASD models. Conversely, in cases of excessive synapse elimination such as SCZ, reducing TREM2 activity could represent a potential therapeutic strategy theoretically. It should be noted, however, that therapies targeting the TREM2 pathway may be influenced by specific physiological contexts and developmental stages, warranting further validation in diverse disease models and human systems. Beyond complement and TREM2, purinergic receptors expressed by microglia, such as P2Y12, are key regulators of process motility, surveillance and basal phagocytic capacity in the healthy brain (127). Experimental work in epilepsy and inflammatory models indicates that selective P2Y12 agonists (e.g., 2-MeSADP) can enhance microglial migration and phagocytic clearance of apoptotic neurons, thereby exerting neuroprotective effects in those contexts (128, 129). These findings raise the speculative possibility that P2Y12R agonism might be harnessed to boost microglia-mediated synaptic engulfment in neurodevelopmental conditions characterized by insufficient pruning. However, to date there is no direct evidence from neurodevelopmental models or clinical studies supporting P2Y12-targeted interventions in ASD, SCZ or related conditions, and such strategies should therefore be regarded as highly hypothetical.

#### 6.1.2 Taming the sentinel: promoting inflammation resolution

Therapeutic strategies targeting neuroinflammation should ideally both suppress the amplification of pro-inflammatory signals and actively promote the resolution of inflammation. At the clinical level, broad-spectrum immunomodulators such as minocycline have shown some, albeit mixed, efficacy in psychiatric cohorts (130), although the available evidence remains limited and largely exploratory. In contrast, targeted precision interventions largely remain at the preclinical stage. The NLRP3 inflammasome may represent an attractive therapeutic target, as it operates upstream in inflammatory signaling and drives the production and release of IL-1β through caspase-1-dependent processing. Evidence from preclinical ASD models indicates that this pathway is dysregulated and closely linked to disease pathology. Selective NLRP3 inhibitors such as MCC950 have been shown to effectively suppress inflammasome activation and attenuate IL-1β-driven pathological damage in these *in vivo* models (92). Studies using murine MIA models further demonstrate that blocking key cytokine pathways can ameliorate neurodevelopmental abnormalities in offspring. For instance, inhibition of IL-17A significantly reduces ASD-like features in MIA-exposed progeny, and interleukin-6 (IL-6) has been identified as a critical mediator linking MIA to aberrant fetal brain development (107, 131).

Beyond suppressing inflammatory responses, highly speculative yet conceptually promising pro-resolving therapies aim to activate signals that facilitate the resolution of inflammation. Bioactive lipids, known as SPMs, can limit further inflammatory amplification and accelerate the non-phlogistic clearance of tissue debris, a process termed efferocytosis. When combined with pro-repair signals such as IL-10, these pathways may safely reprogram microglia toward a homeostatic or tissue-repair-associated state (86).

#### 6.1.3 The pinnacle of modulation: restoring microglial homeostasis

Therapeutic approaches targeting microglia aim to restore homeostasis by modulating microglial states, thereby recovering both synaptic and immune functions. Ideal targets with dual functionality, such as TREM2 and P2Y12R, can regulate microglial phagocytic pruning programs while also influencing their inflammatory transcriptional status (124, 132). However, a major challenge in translating these promising targets into clinical application lies in preserving essential physiological functions while suppressing pathological processes. Broad-spectrum interventions, for instance chronic CSF1R inhibition, may lead to severe depletion of the microglial population and consequently risk the loss of homeostatic surveillance and neurotrophic support, functions that are critical for ongoing brain development (133). Therefore, developing more precise targeting strategies that maximize therapeutic efficacy while minimizing off-target effects and potential adverse consequences represents a key direction worthy of further exploration.

### 6.2 The path forward: challenges and future directions

Although our model offers a unified framework linking microglial dysfunction to the neurodevelopmental continuum, translating these insights into clinical practice faces several formidable challenges. One major obstacle is the need to selectively suppress pathological microglial activity while preserving their essential roles in homeostatic surveillance and neuronal support (134, 135). Another challenge lies in the timing of intervention, as it must occur within specific developmental windows before synaptic and circuit deficits become irreversible, emphasizing the importance of early detection and accurate stratification. Finally, achieving efficient penetration of BBB for precise regional drug delivery remains a major pharmacological hurdle (136–138).

Optimal therapeutic outcomes require us to move beyond single-pathway thinking and adopt a more comprehensive, stage-specific precision strategy. Clinically, biomarkers such as cerebrospinal fluid profiles and neuroimaging readouts may prove invaluable in identifying the optimal intervention window and guiding combination therapies. Mechanistically, coupling the inhibition of upstream inflammatory signals with the activation of downstream pro-resolving pathways could both restrain inflammation and preserve systemic homeostasis. Importantly, recent experimental evidence highlights that non-pharmacological and sensory interventions can directly dictate microglia-dependent synaptic remodeling. For instance, a compelling recent study demonstrated that repetitive unidirectional spinal tactile stimulation actively enhances microglia-mediated synapse remodeling in the medial prefrontal cortex during adolescence (139). This suggests that specific behavioral, sensory, or physical interventions can profoundly shape microglial functions *in vivo*. Moreover, non-invasive approaches leveraging the microbiome-gut-brain axis also offer new avenues for broadly recalibrating the primed state of microglia (120).

Overall, this integrative strategy shifts the focus from symptom management to targeting the underlying pathological cycle, using dynamic biomarkers to facilitate more precise and individualized interventions. Nonetheless, key translational questions remain, including how to establish long-term safety of microglia-targeted modulation and how to balance restoration of homeostasis against preservation of fundamental neural activity and developmental plasticity.

## Data Availability

The original contributions presented in the study are included in the article/supplementary material, Further inquiries can be directed to the corresponding author.

## Glossary

ASD: Autism Spectrum Disorder
BBB: Blood-Brain Barrier
C1q: Complement component 1q
C3: Complement component 3
C4: Complement component 4
CNS: Central Nervous System
CR3: Complement Receptor 3
CSF1R: Colony-Stimulating Factor 1 Receptor
CX3CL1: C-X3-C Motif Chemokine Ligand 1 (also known as Fractalkine)
CX3CR1: C-X3-C Motif Chemokine Receptor 1
DAMPs: Damage-Associated Molecular Patterns
IL-10: Interleukin-10
IL-17A: Interleukin-17A
IL-1β: Interleukin-1 beta
JAK/STAT: Janus Kinase/Signal Transducer and Activator of Transcription
mtDNA: Mitochondrial DNA
MIA: Maternal Immune Activation
NDDs: Neurodevelopmental Disorders
NF-κB: Nuclear Factor-kappa B
PRRs: Pattern Recognition Receptors
SCZ: Schizophrenia
SPMs: Specialized Pro-resolving Mediators
TNF-α: Tumor Necrosis Factor-alpha
TREM2: Triggering Receptor Expressed on Myeloid cells 2

## References

[1] Owen MJ O’Donovan MC . Schizophrenia and the neurodevelopmental continuum: evidence from genomics. World Psychiatry Off J World Psychiatr Assoc. (2017) 16:227–35. doi: 10.1002/wps.20440 28941101 PMC5608820

[2] Global Burden of Disease Study 2021 Autism Spectrum Collaborators . The global epidemiology and health burden of the autism spectrum: findings from the global burden of disease study 2021. Lancet Psychiatry. (2025) 12:111–21. doi: 10.1016/S2215-0366(24)00363-8 39709974 PMC11750762

[3] GBD 2019 Mental Disorders Collaborators . Global, regional, and national burden of 12 mental disorders in 204 countries and territories, 1990-2019: a systematic analysis for the global burden of disease study 2019. Lancet Psychiatry. (2022) 9:137–50. doi: 10.1016/S2215-0366(21)00395-3 35026139 PMC8776563

[4] Yamasaki T Maekawa T Fujita T Tobimatsu S . Connectopathy in autism spectrum disorders: a review of evidence from visual evoked potentials and diffusion magnetic resonance imaging. Front Neurosci. (2017) 11:627. doi: 10.3389/fnins.2017.00627 29170625 PMC5684146

[5] Ni R-J Yuan W-J Wang Y-Y Yang X Wei J-X Zhao L-S . Microglia-mediated inflammation and synaptic pruning contribute to sleep deprivation-induced mania in a sex-specific manner. Transl Psychiatry. (2025) 15:285. doi: 10.1038/s41398-025-03525-x 40817224 PMC12356969

[6] Bagni C Zukin RS . A synaptic perspective of fragile X syndrome and autism spectrum disorders. Neuron. (2019) 101:1070–88. doi: 10.1016/j.neuron.2019.02.041 30897358 PMC9628679

[7] Tang G Gudsnuk K Kuo S-H Cotrina ML Rosoklija G Sosunov A . Loss of mTOR-dependent macroautophagy causes autistic-like synaptic pruning deficits. Neuron. (2014) 83:1131–43. doi: 10.1016/j.neuron.2014.07.040 25155956 PMC4159743

[8] Yilmaz M Yalcin E Presumey J Aw E Ma M Whelan CW . Overexpression of schizophrenia susceptibility factor human complement C4A promotes excessive synaptic loss and behavioral changes in mice. Nat Neurosci. (2021) 24:214–24. doi: 10.1038/s41593-020-00763-8 33353966 PMC8086435

[9] Vasistha NA Sawa A . Prenatal immune stress: its impact on brain development and neuropsychiatric disorders. Annu Rev Neurosci. (2025) 48:345–61. doi: 10.1146/annurev-neuro-112723-024048 40198846

[10] Gangadin SS Enthoven AD van Beveren NJM Laman JD Sommer IEC . Immune dysfunction in schizophrenia spectrum disorders. Annu Rev Clin Psychol. (2024) 20:229–57. doi: 10.1146/annurev-clinpsy-081122-013201 38996077

[11] Fumagalli L Nazlie Mohebiany A Premereur J Polanco Miquel P Bijnens B Van de Walle P . Microglia heterogeneity, modeling and cell-state annotation in development and neurodegeneration. Nat Neurosci. (2025) 28:1381–92. doi: 10.1038/s41593-025-01931-4 40195564

[12] Chen Y Du X Zhang X Li F Yuan S Wang W . Research trends of inflammation in autism spectrum disorders: a bibliometric analysis. Front Immunol. (2025) 16:1534660. doi: 10.3389/fimmu.2025.1534660 40028326 PMC11868081

[13] Hartmann S-M Heider J Wüst R Fallgatter AJ Volkmer H . Microglia-neuron interactions in schizophrenia. Front Cell Neurosci. (2024) 18:1345349. doi: 10.3389/fncel.2024.1345349 38510107 PMC10950997

[14] Mordelt A de Witte LD . Microglia-mediated synaptic pruning as a key deficit in neurodevelopmental disorders: hype or hope? Curr Opin Neurobiol. (2023) 79:102674. doi: 10.1016/j.conb.2022.102674 36657237

[15] Schafer DP Lehrman EK Kautzman AG Koyama R Mardinly AR Yamasaki R . Microglia sculpt postnatal neural circuits in an activity and complement-dependent manner. Neuron. (2012) 74:691–705. doi: 10.1016/j.neuron.2012.03.026 22632727 PMC3528177

[16] Teter OM McQuade A Hagan V Liang W Dräger NM Sattler SM . CRISPRi-based screen of autism spectrum disorder risk genes in microglia uncovers roles of ADNP in microglia endocytosis and synaptic pruning. Mol Psychiatry. (2025) 30:4176–93. doi: 10.1038/s41380-025-02997-z 40188316 PMC12339388

[17] Petry P Oschwald A Merkt S Dinh T-L Andrieux G Crisand C . Early microglia progenitors colonize the embryonic CNS via integrin-mediated migration from the pial surface. Dev Cell. (2025) S1534-5807:532–5. doi: 10.1016/j.devcel.2025.08.012 40934928

[18] Ginhoux F Greter M Leboeuf M Nandi S See P Gokhan S . Fate mapping analysis reveals that adult microglia derive from primitive macrophages. Science. (2010) 330:841–5. doi: 10.1126/science.1194637 20966214 PMC3719181

[19] Elmore MRP Najafi AR Koike MA Dagher NN Spangenberg EE Rice RA . Colony-stimulating factor 1 receptor signaling is necessary for microglia viability, unmasking a microglia progenitor cell in the adult brain. Neuron. (2014) 82:380–97. doi: 10.1016/j.neuron.2014.02.040 24742461 PMC4161285

[20] Walker DG Tang TM Lue L-F . Studies on colony stimulating factor receptor-1 and ligands colony stimulating factor-1 and interleukin-34 in alzheimer’s disease brains and human microglia. Front Aging Neurosci. (2017) 9:244. doi: 10.3389/fnagi.2017.00244 28848420 PMC5552759

[21] Hagemeyer N Hanft K-M Akriditou M-A Unger N Park ES Stanley ER . Microglia contribute to normal myelinogenesis and to oligodendrocyte progenitor maintenance during adulthood. Acta Neuropathol (Berl). (2017) 134:441–58. doi: 10.1007/s00401-017-1747-1 28685323 PMC5951721

[22] Kierdorf K Erny D Goldmann T Sander V Schulz C Perdiguero EG . Microglia emerge from erythromyeloid precursors via Pu.1- and Irf8-dependent pathways. Nat Neurosci. (2013) 16:273–80. doi: 10.1038/nn.3318 23334579

[23] Kim H-G Berdasco C Nairn AC Kim Y . The WAVE complex in developmental and adulthood brain disorders. Exp Mol Med. (2025) 57:13–29. doi: 10.1038/s12276-024-01386-w 39774290 PMC11799376

[24] Bishnoi IR Bordt EA . Sex and region-specific differences in microglial morphology and function across development. Neuroglia Basel Switz. (2025) 6:2. doi: 10.3390/neuroglia6010002 40181886 PMC11967618

[25] Groh J Feng R Yuan X Liu L Klein D Hutahaean G . Microglia activation orchestrates CXCL10-mediated CD8+ T cell recruitment to promote aging-related white matter degeneration. Nat Neurosci. (2025) 28:1160–73. doi: 10.1038/s41593-025-01955-w 40404995 PMC12148934

[26] Ransohoff RM . A polarizing question: do M1 and M2 microglia exist? Nat Neurosci. (2016) 19:987–91. doi: 10.1038/nn.4338 27459405

[27] Berki P Cserép C Környei Z Pósfai B Szabadits E Domonkos A . Microglia contribute to neuronal synchrony despite endogenous ATP-related phenotypic transformation in acute mouse brain slices. Nat Commun. (2024) 15:5402. doi: 10.1038/s41467-024-49773-1 38926390 PMC11208608

[28] Durán Laforet V Schafer DP . Microglia: activity-dependent regulators of neural circuits. Ann N Y Acad Sci. (2024) 1533:38–50. doi: 10.1111/nyas.15105 38294960 PMC10976428

[29] Keren-Shaul H Spinrad A Weiner A Matcovitch-Natan O Dvir-Szternfeld R Ulland TK . A unique microglia type associated with restricting development of alzheimer’s disease. Cell. (2017) 169:1276–1290.e17. doi: 10.1016/j.cell.2017.05.018 28602351

[30] Martins-Ferreira R Calafell-Segura J Leal B Rodríguez-Ubreva J Martínez-Saez E Mereu E . The human microglia atlas (HuMicA) unravels changes in disease-associated microglia subsets across neurodegenerative conditions. Nat Commun. (2025) 16:739. doi: 10.1038/s41467-025-56124-1 39820004 PMC11739505

[31] Escoubas CC Dorman LC Nguyen PT Lagares-Linares C Nakajo H Anderson SR . Type-I-interferon-responsive microglia shape cortical development and behavior. Cell. (2024) 187:1936–1954.e24. doi: 10.1016/j.cell.2024.02.020 38490196 PMC11015974

[32] Zhao Q Wang Q Wang J Tang M Huang S Peng K . Maternal immune activation-induced PPARγ-dependent dysfunction of microglia associated with neurogenic impairment and aberrant postnatal behaviors in offspring. Neurobiol Dis. (2019) 125:1–13. doi: 10.1016/j.nbd.2019.01.005 30659984

[33] Han VX Patel S Jones HF Dale RC . Maternal immune activation and neuroinflammation in human neurodevelopmental disorders. Nat Rev Neurol. (2021) 17:564–79. doi: 10.1038/s41582-021-00530-8 34341569

[34] Paolicelli RC Bolasco G Pagani F Maggi L Scianni M Panzanelli P . Synaptic pruning by microglia is necessary for normal brain development. Science. (2011) 333:1456–8. doi: 10.1126/science.1202529 21778362

[35] Tieu T Cruz A-J Weinstein JR Shih AY Coelho-Santos V . Physiological and injury-induced microglial dynamics across the lifespan. Cell Rep. (2025) 44:115991. doi: 10.1016/j.celrep.2025.115991 40668678 PMC12433668

[36] Huo A Wang J Li Q Li M Qi Y Yin Q . Molecular mechanisms underlying microglial sensing and phagocytosis in synaptic pruning. Neural Regener Res. (2024) 19:1284–90. doi: 10.4103/1673-5374.385854 37905877 PMC11467947

[37] Stevens B Allen NJ Vazquez LE Howell GR Christopherson KS Nouri N . The classical complement cascade mediates CNS synapse elimination. Cell. (2007) 131:1164–78. doi: 10.1016/j.cell.2007.10.036 18083105

[38] Lehrman EK Wilton DK Litvina EY Welsh CA Chang ST Frouin A . CD47 protects synapses from excess microglia-mediated pruning during development. Neuron. (2018) 100:120–134.e6. doi: 10.1016/j.neuron.2018.09.017 30308165 PMC6314207

[39] Nishi Y Toritsuka M Takada R Ishikawa M Ishida R Kayashima Y . Impaired synaptosome phagocytosis in macrophages of individuals with autism spectrum disorder. Mol Psychiatry. (2025) 30:3837–45. doi: 10.1038/s41380-025-03002-3 40185900 PMC12240830

[40] Tremblay M-È Lowery RL Majewska AK . Microglial interactions with synapses are modulated by visual experience. PloS Biol. (2010) 8:e1000527. doi: 10.1371/journal.pbio.1000527 21072242 PMC2970556

[41] Nagappan-Chettiar S Burbridge TJ Umemori H . Activity-dependent synapse refinement: from mechanisms to molecules. Neurosci Rev J Bringing Neurobiol Neurol Psychiatry. (2024) 30:673–89. doi: 10.1177/10738584231170167 37140155 PMC11584027

[42] Scott-Hewitt N Perrucci F Morini R Erreni M Mahoney M Witkowska A . Local externalization of phosphatidylserine mediates developmental synaptic pruning by microglia. EMBO J. (2020) 39:e105380. doi: 10.15252/embj.2020105380 32657463 PMC7429741

[43] Weinhard L di Bartolomei G Bolasco G MaChado P Schieber NL Neniskyte U . Microglia remodel synapses by presynaptic trogocytosis and spine head filopodia induction. Nat Commun. (2018) 9:1228. doi: 10.1038/s41467-018-03566-5 29581545 PMC5964317

[44] Sekar A Bialas AR de Rivera H Davis A Hammond TR Kamitaki N . Schizophrenia risk from complex variation of complement component 4. Nature. (2016) 530:177–83. doi: 10.1038/nature16549 26814963 PMC4752392

[45] Schafer DP Heller CT Gunner G Heller M Gordon C Hammond T . Microglia contribute to circuit defects in Mecp2 null mice independent of microglia-specific loss of Mecp2 expression. eLife. (2016) 5:e15224. doi: 10.7554/eLife.15224 27458802 PMC4961457

[46] Molnár Z Garel S López-Bendito G Maness P Price DJ . Mechanisms controlling the guidance of thalamocortical axons through the embryonic forebrain. Eur J Neurosci. (2012) 35:1573–85. doi: 10.1111/j.1460-9568.2012.08119.x 22607003 PMC4370206

[47] Miyamoto A Wake H Ishikawa AW Eto K Shibata K Murakoshi H . Microglia contact induces synapse formation in developing somatosensory cortex. Nat Commun. (2016) 7:12540. doi: 10.1038/ncomms12540 27558646 PMC5007295

[48] Yu Q Guan T Guo Y Kong J . The initial myelination in the central nervous system. ASN Neuro. (2023) 15:17590914231163039. doi: 10.1177/17590914231163039 36974372 PMC10052612

[49] Chung W-S Clarke LE Wang GX Stafford BK Sher A Chakraborty C . Astrocytes mediate synapse elimination through MEGF10 and MERTK pathways. Nature. (2013) 504:394–400. doi: 10.1038/nature12776 24270812 PMC3969024

[50] Kaiser N Pätz C Brachtendorf S Eilers J Bechmann I . Undisturbed climbing fiber pruning in the cerebellar cortex of CX3 CR1-deficient mice. Glia. (2020) 68:2316–29. doi: 10.1002/glia.23842 32488990

[51] Czeh M Gressens P Kaindl AM . The yin and yang of microglia. Dev Neurosci. (2011) 33:199–209. doi: 10.1159/000328989 21757877

[52] Liu J Liu L Wang X Jiang R Bai Q Wang G . Microglia: A double-edged sword in intracerebral hemorrhage from basic mechanisms to clinical research. Front Immunol. (2021) 12:675660. doi: 10.3389/fimmu.2021.675660 34025674 PMC8135095

[53] Mastenbroek LJM Kooistra SM Eggen BJL Prins JR . The role of microglia in early neurodevelopment and the effects of maternal immune activation. Semin Immunopathol. (2024) 46:1. doi: 10.1007/s00281-024-01017-6 38990389 PMC11239780

[54] Franceschini D Zusso M . Editorial: Neuroinflammation in the developing brain. Int J Dev Neurosci. (2019) 77:1–2. doi: 10.1016/j.ijdevneu.2019.05.007 31152854

[55] Norden DM Godbout JP . Microglia of the aged brain: Primed to be activated and resistant to regulation. Neuropathol Appl Neurobiol. (2013) 39:19–34. doi: 10.1111/j.1365-2990.2012.01306.x 23039106 PMC3553257

[56] Estes ML McAllister AK . Maternal immune activation: Implications for neuropsychiatric disorders. Science. (2016) 353:772–7. doi: 10.1126/science.aag3194 27540164 PMC5650490

[57] Mallard C Tremblay M-E Vexler ZS . Microglia and neonatal brain injury. Neuroscience. (2019) 405:68–76. doi: 10.1016/j.neuroscience.2018.01.023 29352997 PMC6790108

[58] Matsuba Y Nagata K Kadota Y Sahara N Saido TC Hashimoto S . Rod-shaped microglia represent a morphologically distinct subpopulation of disease-associated microglia. J Neuroinflamm. (2025) 22:184. doi: 10.1186/s12974-025-03504-5 40671004 PMC12269120

[59] Zhang Q Wang S-S Zhang Z Chu S-F . PKM2-mediated metabolic reprogramming of microglia in neuroinflammation. Cell Death Discov. (2025) 11:149. doi: 10.1038/s41420-025-02453-5 40189596 PMC11973174

[60] Sadeghdoust M Das A Kaushik DK . Fueling neurodegeneration: Metabolic insights into microglia functions. J Neuroinflamm. (2024) 21:300. doi: 10.1186/s12974-024-03296-0 39551788 PMC11571669

[61] Gao C Jiang J Tan Y Chen S . Microglia in neurodegenerative diseases: Mechanism and potential therapeutic targets. Signal Transduct Target Ther. (2023) 8:359. doi: 10.1038/s41392-023-01588-0 37735487 PMC10514343

[62] Anilkumar S Wright-Jin E . NF-κB as an inducible regulator of inflammation in the central nervous system. Cells. (2024) 13:485. doi: 10.3390/cells13060485 38534329 PMC10968931

[63] Li Y Li Y-J Zhu Z-Q . To re-examine the intersection of microglial activation and neuroinflammation in neurodegenerative diseases from the perspective of pyroptosis. Front Aging Neurosci. (2023) 15:1284214. doi: 10.3389/fnagi.2023.1284214 38020781 PMC10665880

[64] Canovas B Nebreda AR . Diversity and versatility of p38 kinase signalling in health and disease. Nat Rev Mol Cell Biol. (2021) 22:346–66. doi: 10.1038/s41580-020-00322-w 33504982 PMC7838852

[65] Huang S Zhang Y Shu H Liu W Zhou X Zhou X . Advances of the MAPK pathway in the treatment of spinal cord injury. CNS Neurosci Ther. (2024) 30:e14807. doi: 10.1111/cns.14807 38887853 PMC11183187

[66] Hu X Li J Fu M Zhao X Wang W . The JAK/STAT signaling pathway: From bench to clinic. Signal Transduct Target Ther. (2021) 6:402. doi: 10.1038/s41392-021-00791-1 34824210 PMC8617206

[67] Dong L Li Y-Z An H-T Wang Y-L Chen S-H Qian Y-J . The E3 ubiquitin ligase c-cbl inhibits microglia-mediated CNS inflammation by regulating PI3K/akt/NF-κB pathway. CNS Neurosci Ther. (2016) 22:661–9. doi: 10.1111/cns.12557 27156691 PMC6492864

[68] Chu E Mychasiuk R Hibbs ML Semple BD . Dysregulated phosphoinositide 3-kinase signaling in microglia: Shaping chronic neuroinflammation. J Neuroinflamm. (2021) 18:276. doi: 10.1186/s12974-021-02325-6 34838047 PMC8627624

[69] Adamu A Li S Gao F Xue G . The role of neuroinflammation in neurodegenerative diseases: Current understanding and future therapeutic targets. Front Aging Neurosci. (2024) 16:1347987. doi: 10.3389/fnagi.2024.1347987 38681666 PMC11045904

[70] Gagliano A Cucinotta F Giunta I Di Modica I De Domenico C Costanza C . The immune/inflammatory underpinnings of neurodevelopmental disorders and pediatric acute-onset neuropsychiatric syndrome: A scoping review. Int J Mol Sci. (2025) 26:7767. doi: 10.3390/ijms26167767 40869088 PMC12386443

[71] Song X Gao X Wang Y Raja R Zhang Y Yang S . HCV core protein induces chemokine CCL2 and CXCL10 expression through NF-κB signaling pathway in macrophages. Front Immunol. (2021) 12:654998. doi: 10.3389/fimmu.2021.654998 34531848 PMC8438213

[72] Semple BD Kossmann T Morganti-Kossmann MC . Role of chemokines in CNS health and pathology: a focus on the CCL2/CCR2 and CXCL8/CXCR2 networks. J Cereb Blood Flow Metab Off J Int Soc Cereb Blood Flow Metab. (2010) 30:459–73. doi: 10.1038/jcbfm.2009.240 19904283 PMC2949152

[73] Haslund‐Vinding J McBean G Jaquet V Vilhardt F . NADPH oxidases in oxidant production by microglia: Activating receptors, pharmacology and association with disease. Br J Pharmacol. (2017) 174:1733–49. doi: 10.1111/bph.13425 26750203 PMC5446574

[74] Rojo AI McBean G Cindric M Egea J López MG Rada P . Redox control of microglial function: Molecular mechanisms and functional significance. Antioxid Redox Signal. (2014) 21:1766–801. doi: 10.1089/ars.2013.5745 24597893 PMC4186766

[75] Han Q-Q Shen S-Y Liang L-F Chen X-R Yu J . Complement C1q/C3-CR3 signaling pathway mediates abnormal microglial phagocytosis of synapses in a mouse model of depression. Brain Behav Immun. (2024) 119:454–64. doi: 10.1016/j.bbi.2024.04.018 38642614

[76] Chung H-Y Wickel J Hahn N Mein N Schwarzbrunn M Koch P . Microglia mediate neurocognitive deficits by eliminating C1q-tagged synapses in sepsis-associated encephalopathy. Sci Adv. (2023) 9:eabq7806. doi: 10.1126/sciadv.abq7806 37235660 PMC10219600

[77] Sellgren CM Gracias J Watmuff B Biag JD Thanos JM Whittredge PB . Increased synapse elimination by microglia in schizophrenia patient-derived models of synaptic pruning. Nat Neurosci. (2019) 22:374–85. doi: 10.1038/s41593-018-0334-7 30718903 PMC6410571

[78] Niescier RF Lin Y-C . The potential role of AMPA receptor trafficking in autism and other neurodevelopmental conditions. Neuroscience. (2021) 479:180–91. doi: 10.1016/j.neuroscience.2021.09.013 34571086

[79] Cainelli E Arrigoni F Vedovelli L . White matter injury and neurodevelopmental disabilities: A cross-disease (dis)connection. Prog Neurobiol. (2020) 193:101845. doi: 10.1016/j.pneurobio.2020.101845 32505757

[80] Schmierer B Hill CS . TGFbeta-SMAD signal transduction: Molecular specificity and functional flexibility. Nat Rev Mol Cell Biol. (2007) 8:970–82. doi: 10.1038/nrm2297 18000526

[81] Li Q Barres BA . Microglia and macrophages in brain homeostasis and disease. Nat Rev Immunol. (2018) 18:225–42. doi: 10.1038/nri.2017.125 29151590

[82] Lobo-Silva D Carriche GM Castro AG Roque S Saraiva M . Balancing the immune response in the brain: IL-10 and its regulation. J Neuroinflamm. (2016) 13:297. doi: 10.1186/s12974-016-0763-8 27881137 PMC5121946

[83] Huang J-M Zhao N Hao X-N Li S-Y Wei D Pu N . CX3CL1/CX3CR1 signaling mediated neuroglia activation is implicated in the retinal degeneration: A potential therapeutic target to prevent photoreceptor death. Invest Ophthalmol Vis Sci. (2024) 65:29. doi: 10.1167/iovs.65.1.29 38231527 PMC10795588

[84] Teixeira-Santos L Albino-Teixeira A Pinho D . Neuroinflammation, oxidative stress and their interplay in neuropathic pain: focus on specialized pro-resolving mediators and NADPH oxidase inhibitors as potential therapeutic strategies. Pharmacol Res. (2020) 162:105280. doi: 10.1016/j.phrs.2020.105280 33161139

[85] Kooij G Troletti CD Leuti A Norris PC Riley I Albanese M . Specialized pro-resolving lipid mediators are differentially altered in peripheral blood of patients with multiple sclerosis and attenuate monocyte and blood-brain barrier dysfunction. Haematologica. (2020) 105:2056–70. doi: 10.3324/haematol.2019.219519 31780628 PMC7395264

[86] Serhan CN . Pro-resolving lipid mediators are leads for resolution physiology. Nature. (2014) 510:92–101. doi: 10.1038/nature13479 24899309 PMC4263681

[87] Fernández de Cossío L Lacabanne C Bordeleau M Castino G Kyriakakis P Tremblay M-È . Lipopolysaccharide-induced maternal immune activation modulates microglial CX3CR1 protein expression and morphological phenotype in the hippocampus and dentate gyrus, resulting in cognitive inflexibility during late adolescence. Brain Behav Immun. (2021) 97:440–54. doi: 10.1016/j.bbi.2021.07.025 34343619

[88] Ishizuka K Fujita Y Kawabata T Kimura H Iwayama Y Inada T . Rare genetic variants in CX3CR1 and their contribution to the increased risk of schizophrenia and autism spectrum disorders. Transl Psychiatry. (2017) 7:e1184. doi: 10.1038/tp.2017.173 28763059 PMC5611740

[89] Lai W Chen J Zhu H Jiang Y Niu Y Chen Q . The knockout of moesin leads to excess microglia-mediated synaptic pruning and impairs social novelty in a mouse model. Cell Rep. (2025) 44:116012. doi: 10.1016/j.celrep.2025.116012 40674216

[90] Sarn N Jaini R Thacker S Lee H Dutta R Eng C . Cytoplasmic-predominant pten increases microglial activation and synaptic pruning in a murine model with autism-like phenotype. Mol Psychiatry. (2021) 26:1458–71. doi: 10.1038/s41380-020-0681-0 32055008 PMC8159731

[91] Ju J Pan Y Yang X Li X Chen J Wu S . The “don’t eat me” signal CD47 is associated with microglial phagocytosis defects and autism-like behaviors in 16p11.2 deletion mice. Proc Natl Acad Sci USA. (2025) 122:e2411080122. doi: 10.1073/pnas.2411080122 40238451 PMC12036979

[92] Vallese A Cordone V Ferrara F Guiotto A Gemmo L Cervellati F . NLRP3 inflammasome-mitochondrion loop in autism spectrum disorder. Free Radic Biol Med. (2024) 225:581–94. doi: 10.1016/j.freeradbiomed.2024.10.297 39433111

[93] Trujillo Villarreal L- Cárdenas-Tueme M Maldonado-Ruiz R Reséndez-Pérez D Camacho-Morales A . Potential role of primed microglia during obesity on the mesocorticolimbic circuit in autism spectrum disorder. J Neurochem. (2021) 156:415–34. doi: 10.1111/jnc.15141 32902852

[94] Derecki NC Cronk JC Kipnis J . The role of microglia in brain maintenance: Implications for rett syndrome. Trends Immunol. (2013) 34:144–50. doi: 10.1016/j.it.2012.10.002 23122051 PMC3566274

[95] Lin M-M Liu N Qin Z-H Wang Y . Mitochondrial-derived damage-associated molecular patterns amplify neuroinflammation in neurodegenerative diseases. Acta Pharmacol Sin. (2022) 43:2439–47. doi: 10.1038/s41401-022-00879-6 35233090 PMC9525705

[96] Huang S Dong W Lin X Xu K Li K Xiong S . Disruption of the na+/K+-ATPase-purinergic P2X7 receptor complex in microglia promotes stress-induced anxiety. Immunity. (2024) 57:495–512.e11. doi: 10.1016/j.immuni.2024.01.018 38395698

[97] Matuleviciute R Akinluyi ET Muntslag TAO Dewing JM Long KR Vernon AC . Microglial contribution to the pathology of neurodevelopmental disorders in humans. Acta Neuropathol (Berl). (2023) 146:663–83. doi: 10.1007/s00401-023-02629-2 37656188 PMC10564830

[98] Knuesel I Chicha L Britschgi M Schobel SA Bodmer M Hellings JA . Maternal immune activation and abnormal brain development across CNS disorders. Nat Rev Neurol. (2014) 10:643–60. doi: 10.1038/nrneurol.2014.187 25311587

[99] Murao A Aziz M Wang H Brenner M Wang P . Release mechanisms of major DAMPs. Apoptosis Int J Program Cell Death. (2021) 26:152–62. doi: 10.1007/s10495-021-01663-3 33713214 PMC8016797

[100] Fan G Ma J Ma R Suo M Chen Y Zhang S . Microglia modulate neurodevelopment in autism spectrum disorder and schizophrenia. Int J Mol Sci. (2023) 24:17297. doi: 10.3390/ijms242417297 38139124 PMC10743577

[101] Morgan JT Chana G Pardo CA Achim C Semendeferi K Buckwalter J . Microglial activation and increased microglial density observed in the dorsolateral prefrontal cortex in autism. Biol Psychiatry. (2010) 68:368–76. doi: 10.1016/j.biopsych.2010.05.024 20674603

[102] Vargas DL Nascimbene C Krishnan C Zimmerman AW Pardo CA . Neuroglial activation and neuroinflammation in the brain of patients with autism. Ann Neurol. (2005) 57:67–81. doi: 10.1002/ana.20315 15546155

[103] Satterstrom FK Kosmicki JA Wang J Breen MS De Rubeis S An J-Y . Large-scale exome sequencing study implicates both developmental and functional changes in the neurobiology of autism. Cell. (2020) 180:568–584.e23. doi: 10.1016/j.cell.2019.12.036 31981491 PMC7250485

[104] Grove J Ripke S Als TD Mattheisen M Walters RK Won H . Identification of common genetic risk variants for autism spectrum disorder. Nat Genet. (2019) 51:431–44. doi: 10.1038/s41588-019-0344-8 30804558 PMC6454898

[105] Malkova NV Yu CZ Hsiao EY Moore MJ Patterson PH . Maternal immune activation yields offspring displaying mouse versions of the three core symptoms of autism. Brain Behav Immun. (2012) 26:607–16. doi: 10.1016/j.bbi.2012.01.011 22310922 PMC3322300

[106] Xu J Zhao R Yan M Zhou M Liu H Wang X . Sex-specific behavioral and molecular responses to maternal lipopolysaccharide-induced immune activation in a murine model: Implications for neurodevelopmental disorders. Int J Mol Sci. (2024) 25:9885. doi: 10.3390/ijms25189885 39337372 PMC11432365

[107] Choi GB Yim YS Wong H Kim S Kim H Kim SV . The maternal interleukin-17a pathway in mice promotes autism-like phenotypes in offspring. Science. (2016) 351:933–9. doi: 10.1126/science.aad0314 26822608 PMC4782964

[108] Luo Y Lv K Du Z Zhang D Chen M Luo J . Minocycline improves autism-related behaviors by modulating microglia polarization in a mouse model of autism. Int Immunopharmacol. (2023) 122:110594. doi: 10.1016/j.intimp.2023.110594 37441807

[109] Owen MJ Legge SE Rees E Walters JTR O’Donovan MC . Genomic findings in schizophrenia and their implications. Mol Psychiatry. (2023) 28:3638–47. doi: 10.1038/s41380-023-02293-8 37853064 PMC10730422

[110] Gogtay N Giedd JN Lusk L Hayashi KM Greenstein D Vaituzis AC . Dynamic mapping of human cortical development during childhood through early adulthood. Proc Natl Acad Sci USA. (2004) 101:8174–9. doi: 10.1073/pnas.0402680101 15148381 PMC419576

[111] McCarley RW Wible CG Frumin M Hirayasu Y Levitt JJ Fischer IA . MRI anatomy of schizophrenia. Biol Psychiatry. (1999) 45:1099–119. doi: 10.1016/s0006-3223(99)00018-9 10331102 PMC2846838

[112] Glantz LA Lewis DA . Decreased dendritic spine density on prefrontal cortical pyramidal neurons in schizophrenia. Arch Gen Psychiatry. (2000) 57:65–73. doi: 10.1001/archpsyc.57.1.65 10632234

[113] Druart M Nosten-Bertrand M Poll S Crux S Nebeling F Delhaye C . Elevated expression of complement C4 in the mouse prefrontal cortex causes schizophrenia-associated phenotypes. Mol Psychiatry. (2021) 26:3489–501. doi: 10.1038/s41380-021-01081-6 33837272

[114] Howes OD Shatalina E . Integrating the neurodevelopmental and dopamine hypotheses of schizophrenia and the role of cortical excitation-inhibition balance. Biol Psychiatry. (2022) 92:501–13. doi: 10.1016/j.biopsych.2022.06.017 36008036

[115] Beopoulos A Géa M Fasano A Iris F . Autism spectrum disorders pathogenesis: Toward a comprehensive model based on neuroanatomic and neurodevelopment considerations. Front Neurosci. (2022) 16:988735. doi: 10.3389/fnins.2022.988735 36408388 PMC9671112

[116] Howes OD Onwordi EC . The synaptic hypothesis of schizophrenia version III: A master mechanism. Mol Psychiatry. (2023) 28:1843–56. doi: 10.1038/s41380-023-02043-w 37041418 PMC10575788

[117] Lynch MA . Exploring sex-related differences in microglia may be a game-changer in precision medicine. Front Aging Neurosci. (2022) 14:868448. doi: 10.3389/fnagi.2022.868448 35431903 PMC9009390

[118] Lenz KM McCarthy MM . A starring role for microglia in brain sex differences. Neurosci Rev J Bringing Neurobiol Neurol Psychiatry. (2015) 21:306–21. doi: 10.1177/1073858414536468 24871624 PMC5742269

[119] Magni G Riboldi B Petroni K Ceruti S . Flavonoids bridging the gut and the brain: Intestinal metabolic fate, and direct or indirect effects of natural supporters against neuroinflammation and neurodegeneration. Biochem Pharmacol. (2022) 205:115257. doi: 10.1016/j.bcp.2022.115257 36179933

[120] Taniya MA Chung H-J Al Mamun A Alam S Aziz MA Emon NU . Role of gut microbiome in autism spectrum disorder and its therapeutic regulation. Front Cell Infect Microbiol. (2022) 12:915701. doi: 10.3389/fcimb.2022.915701 35937689 PMC9355470

[121] Germann M Brederoo SG Sommer IEC . Abnormal synaptic pruning during adolescence underlying the development of psychotic disorders. Curr Opin Psychiatry. (2021) 34:222–7. doi: 10.1097/YCO.0000000000000696 33560023 PMC8048735

[122] Dalakas MC Alexopoulos H Spaeth PJ . Complement in neurological disorders and emerging complement-targeted therapeutics. Nat Rev Neurol. (2020) 16:601–17. doi: 10.1038/s41582-020-0400-0 33005040 PMC7528717

[123] Lansita JA Mease KM Qiu H Yednock T Sankaranarayanan S Kramer S . Nonclinical development of ANX005: A humanized anti-C1q antibody for treatment of autoimmune and neurodegenerative diseases. Int J Toxicol. (2017) 36:449–62. doi: 10.1177/1091581817740873 29202623

[124] Filipello F Morini R Corradini I Zerbi V Canzi A Michalski B . The microglial innate immune receptor TREM2 is required for synapse elimination and normal brain connectivity. Immunity. (2018) 48:979–991.e8. doi: 10.1016/j.immuni.2018.04.016 29752066

[125] Jay TR von Saucken VE Muñoz B Codocedo JF Atwood BK Lamb BT . TREM2 is required for microglial instruction of astrocytic synaptic engulfment in neurodevelopment. Glia. (2019) 67:1873–92. doi: 10.1002/glia.23664 31265185

[126] Cantoni C Bollman B Licastro D Xie M Mikesell R Schmidt R . TREM2 regulates microglial cell activation in response to demyelination *in vivo*. Acta Neuropathol (Berl). (2015) 129:429–47. doi: 10.1007/s00401-015-1388-1 25631124 PMC4667728

[127] Koizumi S Ohsawa K Inoue K Kohsaka S . Purinergic receptors in microglia: Functional modal shifts of microglia mediated by P2 and P1 receptors. Glia. (2013) 61:47–54. doi: 10.1002/glia.22358 22674620

[128] Sunkaria A Bhardwaj S Halder A Yadav A Sandhir R . Migration and phagocytic ability of activated microglia during post-natal development is mediated by calcium-dependent purinergic signalling. Mol Neurobiol. (2016) 53:944–54. doi: 10.1007/s12035-014-9064-3 25575683

[129] Yang X Cao Q Guo Y He J Xu D Lin A . GSDMD knockdown attenuates phagocytic activity of microglia and exacerbates seizure susceptibility in TLE mice. J Neuroinflamm. (2023) 20:193. doi: 10.1186/s12974-023-02876-w 37612735 PMC10464294

[130] Panizzutti B Skvarc D Lin S Croce S Meehan A Bortolasci CC . Minocycline as treatment for psychiatric and neurological conditions: A systematic review and meta-analysis. Int J Mol Sci. (2023) 24:5250. doi: 10.3390/ijms24065250 36982324 PMC10049047

[131] Smith SEP Li J Garbett K Mirnics K Patterson PH . Maternal immune activation alters fetal brain development through interleukin-6. J Neurosci Off J Soc Neurosci. (2007) 27:10695–702. doi: 10.1523/JNEUROSCI.2178-07.2007 17913903 PMC2387067

[132] Suzuki T Kohyama K Moriyama K Ozaki M Hasegawa S Ueno T . Extracellular ADP augments microglial inflammasome and NF-κB activation via the P2Y12 receptor. Eur J Immunol. (2020) 50:205–19. doi: 10.1002/eji.201848013 31549730

[133] Green KN Crapser JD Hohsfield LA . To kill a microglia: A case for CSF1R inhibitors. Trends Immunol. (2020) 41:771–84. doi: 10.1016/j.it.2020.07.001 32792173 PMC7484341

[134] Tremblay M-È . Microglial functional alteration and increased diversity in the challenged brain: Insights into novel targets for intervention. Brain Behav Immun - Health. (2021) 16:100301. doi: 10.1016/j.bbih.2021.100301 34589793 PMC8474548

[135] Aldrich A Kielian T . Central nervous system fibrosis is associated with fibrocyte-like infiltrates. Am J Pathol. (2011) 179:2952–62. doi: 10.1016/j.ajpath.2011.08.036 22015460 PMC3260835

[136] Thion MS Ginhoux F Garel S . Microglia and early brain development: An intimate journey. Science. (2018) 362:185–9. doi: 10.1126/science.aat0474 30309946

[137] Qin J Ma Z Chen X Shu S . Microglia activation in central nervous system disorders: A review of recent mechanistic investigations and development efforts. Front Neurol. (2023) 14:1103416. doi: 10.3389/fneur.2023.1103416 36959826 PMC10027711

[138] Wu D Chen Q Chen X Han F Chen Z Wang Y . The blood-brain barrier: Structure, regulation, and drug delivery. Signal Transduct Target Ther. (2023) 8:217. doi: 10.1038/s41392-023-01481-w 37231000 PMC10212980

[139] Chen Y-N Zhao S-T Hu M-A Li W Yu J Ma M-J . Repetitive unidirectional spinal tactile stimulation engages microglial Bmal1 pathways to promote synaptic remodeling in the mPFC of adolescent VPA-exposed mice. J Neuroinflamm. (2025) 23:23. doi: 10.1186/s12974-025-03627-9 41382128 PMC12822062

